# Explanation of clustering result based on multi-objective optimization

**DOI:** 10.1371/journal.pone.0292960

**Published:** 2023-10-27

**Authors:** Liang Chen, Caiming Zhong, Zehua Zhang

**Affiliations:** 1 Faculty of Electrical Engineering and Computer Science, Ningbo University, Ningbo, Zhejiang, China; 2 College of Science and Technology, Ningbo University, Ningbo, Zhejiang, China; 3 College of Information and Computer, Taiyuan University of Technology, Jinzhong, Shanxi, China; National Kaohsiung University of Science and Technology / Industrial University of Ho Chi Minh, TAIWAN

## Abstract

Clustering is an unsupervised machine learning technique whose goal is to cluster unlabeled data. But traditional clustering methods only output a set of results and do not provide any explanations of the results. Although in the literature a number of methods based on decision tree have been proposed to explain the clustering results, most of them have some disadvantages, such as too many branches and too deep leaves, which lead to complex explanations and make it difficult for users to understand. In this paper, a hypercube overlay model based on multi-objective optimization is proposed to achieve succinct explanations of clustering results. The model designs two objective functions based on the number of hypercubes and the compactness of instances and then uses multi-objective optimization to find a set of nondominated solutions. Finally, an Utopia point is defined to determine the most suitable solution, in which each cluster can be covered by as few hypercubes as possible. Based on these hypercubes, an explanations of each cluster is provided. Upon verification on synthetic and real datasets respectively, it shows that the model can provide a concise and understandable explanations to users.

## Introduction

With the widespread use of Artificial intelligence (AI) technology, AI models have received more and more controversies in society, where the most important aspect lies in their lack of transparency and explainability [1]. Since most AI models are black boxes, users and experts can not understand their logic inside or verify their decision-making process. This drawback will destroy the trust of users in AI models and hinder their application and development in certain fields [2]. Explainability of AI models is not necessarily required in some low-risk fields where incorrect prediction results will not cause serious effects [3]. However, in recent years, AI models have been widely used in fields with high risks and high impact, such as healthcare, criminal justice, and some other regulated fields. Because decision-making in high-risk fields is rather complex and with great impact on society, it is an extremely dangerous decision to leave this important decision-making to a model where humans cannot explain its working logic and understand the decision-making process. In this aspect, the transparency and explainability of AI models are a requisite for such decision-makings. Due to the demand for model explainability in these fields, Explainable Artificial Intelligence (XAI) has gradually become a new research field whose goal is to create a set of XAI models or methods to achieve transparency of the logic behind AI models [4–7]. In recent years, the explainability of Machine Learning (ML) has gradually become a more specific research direction in XAI. In the practice, the lack of explainability means that the model cannot be fully trusted, which leads to unsuccessful application of most data-driven models [8, 9]. In other words, the trust in models can be regarded as a prerequisite for their successful application in practice. Therefore, explainability is an essential feature of ML models, as users or experts need to know and understand how the input data affects the results of model decision-making. [10, 11].

Clustering is the task of grouping a set of objects in such a way that objects in the same group (called a cluster) are more similar (in some sense) to each other than to those in other groups (clusters) [12]. It is a main task of exploratory data analysis, and a common technique for statistical data analysis. In recent years, clustering has been also used in high-risk and high-impact fields like infrastructure development [13], criminal justice [14], and healthcare [15], etc. In these fields, decision makers need to know not only the characteristics of and differences among clusters, but also the specific clustering process, which makes explainability essential for clustering techniques. In addition, the explainability of clustering techniques can help decision makers evaluate the fairness of systems and decide when to trust them. For example, in healthcare applications, the explainability of clustering can provide detailed explanations for the decisions of medical researchers, thus winning the trust of patients and providing a basis for the division of responsibilities [16]. Currently, there are generally two methods to achieve explainable clustering, intrinsic models and post-hoc models. The intrinsic models provide clustering and corresponding explainability simultaneously, while the post-hoc model focuses on explaining the clustering results obtained through a clustering method.

Post-hoc models can be applied to the output of any clustering method. A common method is to simply use an explainable supervised learning algorithm to predict clustering labels, whereby the explanations for clustering is generated based on the explainability of supervised learning [17–19]. Much of the work is to achieve explainability of clustering through the decision tree algorithms [20], and the main contribution of this kind of method is the proposal of a new decision tree splitting criterion. Bertsimas et al. [21] expressed the finding of the optimal decision tree as an optimization problem, and found an approximate solution through coordinate descent. Eduardo Laber et al. [22] propose an efficient algorithm with a penalty term in its loss function to favor the construction of shallow decision trees. The significant difference from the other decision tree based methods is that the metrics related to the depths of the leaves are considered. Another post-hoc method is to find a representative prototype or feature set for each cluster. A common method is to check some statistical data of the corresponding characteristics such as the average, median, or majority. Carrisoza et al. [23] proposed an integer programming formula to find the best prototype and radius that cover all instances in the cluster. Then, the optimal explanations is achieved by weighing the false positive and negative instances.

Intrinsic models integrate explanations and clustering together. Liu et al. [24] used synthetic data points to expand the datasets and transformed clustering into supervised learning. Then, the proposed method is used to classify the original data from the synthetic data and finally obtained a decision tree. Kim et al. [25] proposed an explainable clustering model where a logical formula is used to describe each cluster. The model focuses on binary data but the threshold value of continuous features cannot be automatically determined.

In some other work, the explainability of clustering is achieved by constructing rules. Pellegg et al. [26] proposed a probabilistic generation model of soft rectangles, without involving feature selection. Based on the soft clustering method, Chen et al. [27] proposed a Discriminative Rectangular Mixture (DReaM) model. One of its advantages is that two types of features can be specified randomly, the features of rule generation and the features of cluster structure preservation. In DReaM model, the features of rule generation are used to generate explanations for clustering, while those of cluster structure preservation are used for completing clustering tasks. Furthermore, the model also allows combination of priori knowledge to adjust the distribution of rectangular decision boundaries. Wang et al. [28] also proposed a rule-based soft clustering method. In this method, the clustering task is completed through the changed triangular membership function. What’s more, the generation rules of each cluster can be extracted with the proposed fuzzy division method. This method makes fuzzy division more flexible, but fails to make the sum of the membership of all fuzzy sets 1, which may affect its explainability. Mansoori et al. [29] also used the triangular membership function as the division method. Differing from [28], they added randomly generated auxiliary data into the raw dataset, transforming the clustering problem into a classification problem. Then, this algorithm is used to extract the clustering rules with minimum clustering losses, and the extraction is repeated after removal of auxiliary samples until the stop conditions are meet.

In this paper, a post-hoc explanation method known as Hypercube Overlay Model (HcubeOM) is proposed. HcubeOM first determines the most suitable hypercube overlay scheme for each cluster through multi-objective optimization, and then synthesizes the features of the hypercubes in each cluster to generate explanations for the cluster. The explanations obtained with HcubeOM can visually present the characteristics of each cluster and well explain the differences between clusters.

The main contributions of this paper are as follows.

- An explanation framework for clustering results is designed, which depends on multi-objective optimization. In the framework, multiple hypercubes are used to overlay the results of each cluster, and the explanations for each cluster is generated by integrating the features of the hypercubes therein.
- Two objective functions are designed to determine the hypercube overlay scheme of each cluster, one is to determine the optimal number of hypercubes, and the other is to determine the optimal hypercubes. The optimal hypercubes require that the instances in the hypercube should be as compact as possible while the overlaps among the hypercubes be as small as possible.
- A succinctness metric is designed to verify the performance of the generated explanations for each cluster. Based on the number of hypercubes and the compactness of the instances, a succinctness metric is designed as to evaluate the performance of explanations.

The rest of this paper is organized as follows. Section Related Work briefly describes the related work about multi-objective optimization problem and NSGA-II algorithm. Section The Proposed method presents the HcubeOM method in detail. A comprehensive set of experimental results are provided in Section Experimental Result. Finally, we conclude in Section Summary.

## Related work

### Multi-objective optimization problem

The multi-objective optimization problem is about solving the decision vector that meets specific conditions. The solution vector should not only optimize the function vector composed of several objective functions, but may also be constrained by several variables. As the multi-objective optimization problem naturally faces function vectors that contain more than one objective, the difficulty is that the objective function vectors are usually irreconcilable, which means that one or more feasible solutions should be found in multi-objective optimization to maximize the satisfaction of many optimization objectives. Eq 1 shows the minimized unconstrained optimization problem of *m* objectives without constraint.

$$\begin{array}{c} \begin{array}{c} \text{Min}\;F\left(x\right)=\{f_{1}\left(x\right),f_{2}\left(x\right),\ldots ,f_{m}\left(x\right)\}\\ x=(x_{1},x_{2},\ldots ,x_{i},\ldots ,x_{n})\in \Omega \end{array} \end{array}$$ (1)

where *F*(*x*) = {*f*_1_(*x*), *f*_2_(*x*), …, *f*_*m*_(*x*)} is vector of objective functions to be optimized, Ω means the search space, *m* means the number of objective functions, and *x* means the decision vector composed of *n* decision variables.

When *m* = 1, the above model is simplified to a single-objective optimization problem, where the optimal solution is the decision vector that minimizes the single-objective problem. When *m* > 1, the above model is a multi-objective optimization problem, where the solution needs to be a trade-off between decision vectors that meet *m* conflicting objectives.

### NSGA-II algorithm

**Fig 1:**
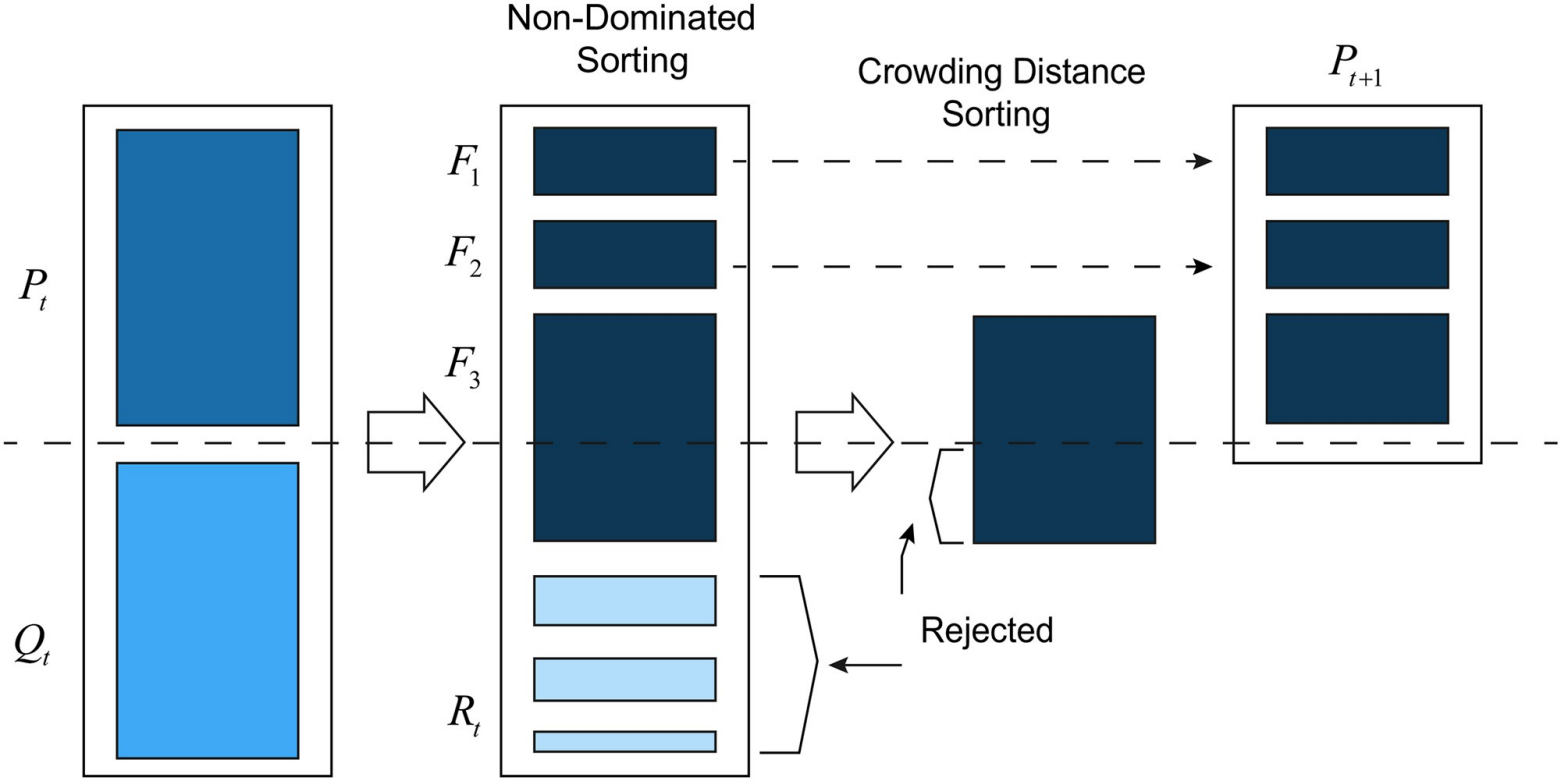
NSGA-II working procedure [31].

NSGA-II [30] is a fairly effective and popular algorithm for solving multi-objective problems, with fast non-dominant sorting as its greatest highlight. On the one hand, this method reduces the calculation complexity, and on the other hand, it also merges the parent population and the child population, so that the next generation is selected from the doubled space and all outstanding individuals are retained. The working procedure of NSGA-II algorithm is shown in Fig 1. First, the population *P*_*t*_ undergoes selection, crossover, and mutation to form population *Q*_*t*_. Then, the two populations are merged into the population *R*_*t*_. Next, population *R*_*t*_ is subject to non-dominated sorting to form multiple fronts *F*_*g*_, and a new generation of population *P*_*t*+1_ is added from low to high. When the addition of *F*_*g*_ causes the population to exceed the size, the individuals are added to the new generation of population *P*_*t*+1_ from large to small based on the crowding distance. Algorithm 1 shows the specific steps of the algorithm.

**Algorithm 1** NSGA-II [30]

**Input:** an initial population *P*_0_, the number of population *pop*

**Output:** a set of optimal solutions *F*_1_

1: *P*_0_ = (*F*_1_, *F*_2_, …) = set non-dominated-sort (*P*_0_)

2: **for** (*F*_*g*_ ∈ *P*_0_) **do**

3:  crowding-distance-assignment(*F*_*g*_)

4: **end for**

5: set *t* = 0

6: **while** loop condition **do**

7:  generate child population *Q*_*t*_ from *P*_*t*_

8:  *R*_*t*_ = *P*_*t*_ ∪ *Q*_*t*_

9:  *F* = fast-nondominated-sort (*R*_*t*_)

10:  *P*_*t*+1_ = ∅

11:  *i* = 1

12:  **while** (∣*P*_*t*+1_∣ + ∣*F*_*g*_∣ < *pop*) **do**

13:   *P*_*t*+1_ = *P*_*t*+1_ ∪ *F*_*g*_

14:   *i* = *i* + 1

15:  **end while**

16:  sort *F*_*g*_ on crowding distance

17:  set *P*_*t*+1_ = *P*_*t*+1_ ∪ *F*_*g*_[1 : (*pop*− ∣*P*_*t*+1_∣)]

18:  set *t* = *t* + 1

19: **end while**

20: **return** *F*_1_

#### Fast-nondominated-sort

In NSGA-II, based on the information of non-domination the individual solutions are sorted on each front. If all other solutions do not dominate on the particular solution, that solution is considered as the first front. If the solution is only dominated by the front one’s solutions that solution is taken as a second front and so on. Here, the domination of solution *S*_1_ over *S*_2_ is determined by considering the following conditions such as if there are *M* objective functions, (1) for all the *M* objective function, the quality of solution *S*_1_ is not always worse than *S*_2_ solution or, (2) *S*_1_ solution is strictly better than *S*_2_ solution in at least one of the *M* objective functions. Solution’s ranking is assigned using the information of the solution’s front number to which the solutions belong. The process of identifying and ranking the non-dominated solutions in Population *GP*_*s*_ are described in Algorithm 2 [31].

**Algorithm 2** Fast-nondominated-sort

**Input:** A set of solutions, *GP*_*s*_.

**Output:** The ranked non-dominated solutions.

 1: Set rank counter *r*_*s*_ = 0.

 2: Increase *r*_*s*_ = *r*_*s*_ + 1.

 3: Identify the non-dominated solutions for the non-dominated front from the set of solutions *GP*_*s*_.

 4: Assign rank *r*_*s*_ to these non-dominated solutions.

 5: Remove these non-dominated solutions from the solution set, *GP*_*s*_.

 6: Go to line 2 until the solution set *GP*_*s*_ = ∅.

#### Crowding distance

**Fig 2:**
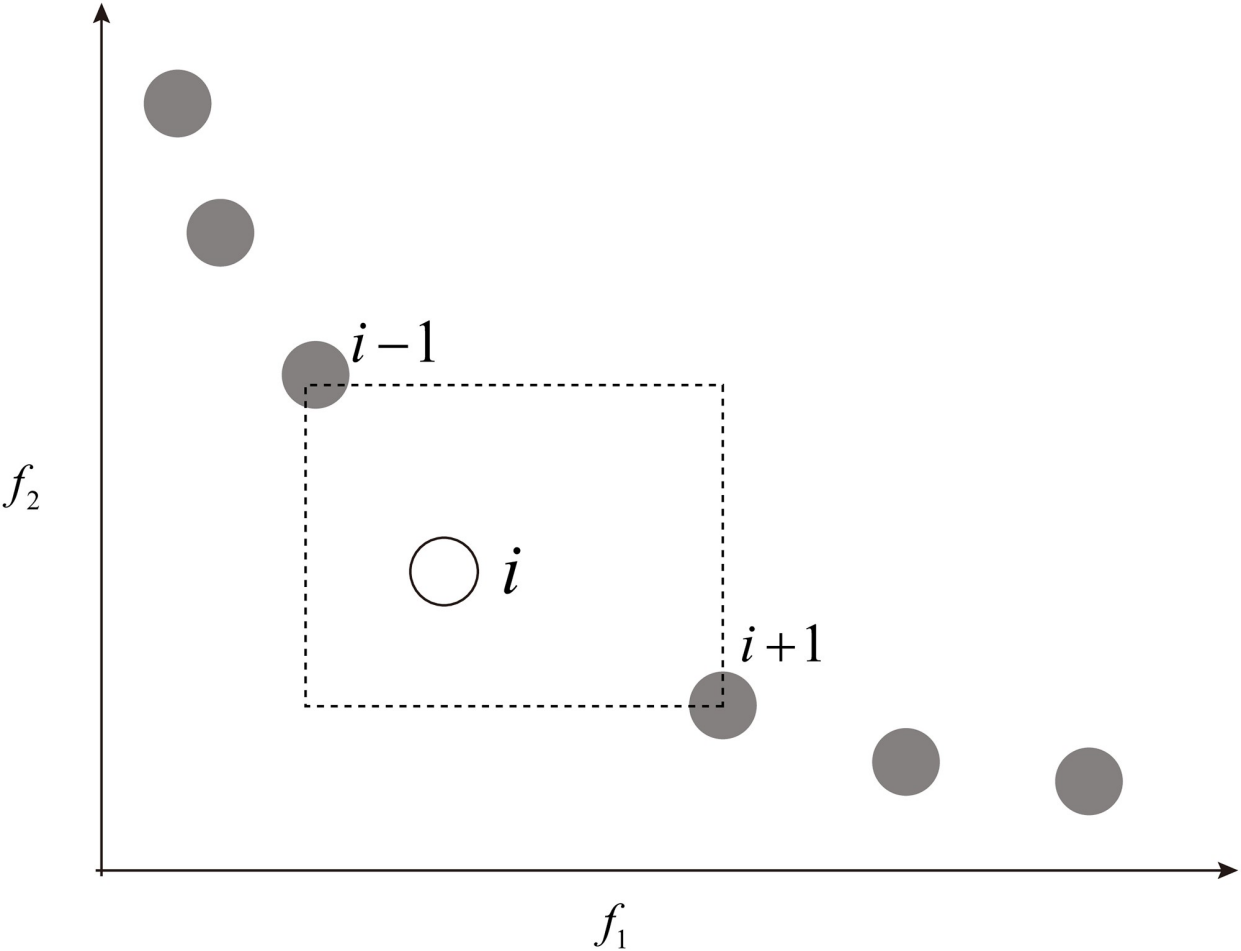
Crowding distance. Crowding distance calculation for the *i*th solution [31].

The crowding-distance computation is a part of density estimation of solutions surrounding an individual solution. For density estimation, it calculates the average distance between two points on either side of this point with each of the objectives. This measure is called crowding distance. Considering the nearest neighbors as the vertices, the crowding distance is treated as a perimeter of the cuboid formed. In Fig 2, the crowding distance of the *i*th solution in its front is the average side length of the cuboid. Suppose, *N* number of non-dominated solutions in *N*_*s*_ set and *M* number of objective functions *Ob*_*i*_, *i* = 1, 2…, *M* are given. Let *dist*_*s*_ denotes the crowding distance value on the solution *s*. The crowding-distance computing process can be outlined by Algorithm 3 [31].

**Algorithm 3** Crowding distance

**Input:** A non-dominated solution set, *N*_*s*_.

**Output:** The crowding distance, *dist*.

 1: Set distance *dist*_*s*_ = 0 for *s* = 1, 2, 3…, *N*.

 2: Sort the solution set *N*_*s*_ for each objective function *Ob*_*i*_, *i* = 1, 2…, *M*.

 3: Set *dist*_1_ = *dist*_*N*_ = ∞(an infinite distance value).

 4: Set $dist_{s}=dist_{s}+\left(Ob_{i(k+1)}-Ob_{i(k-1)}\right)/\left(Ob_{i}^{max}-Ob_{i}^{min}\right)$, for *k* = 2 to (*N* − 1).

 5: **return** *dist*_*s*_

## The proposed model

**Fig 3:**
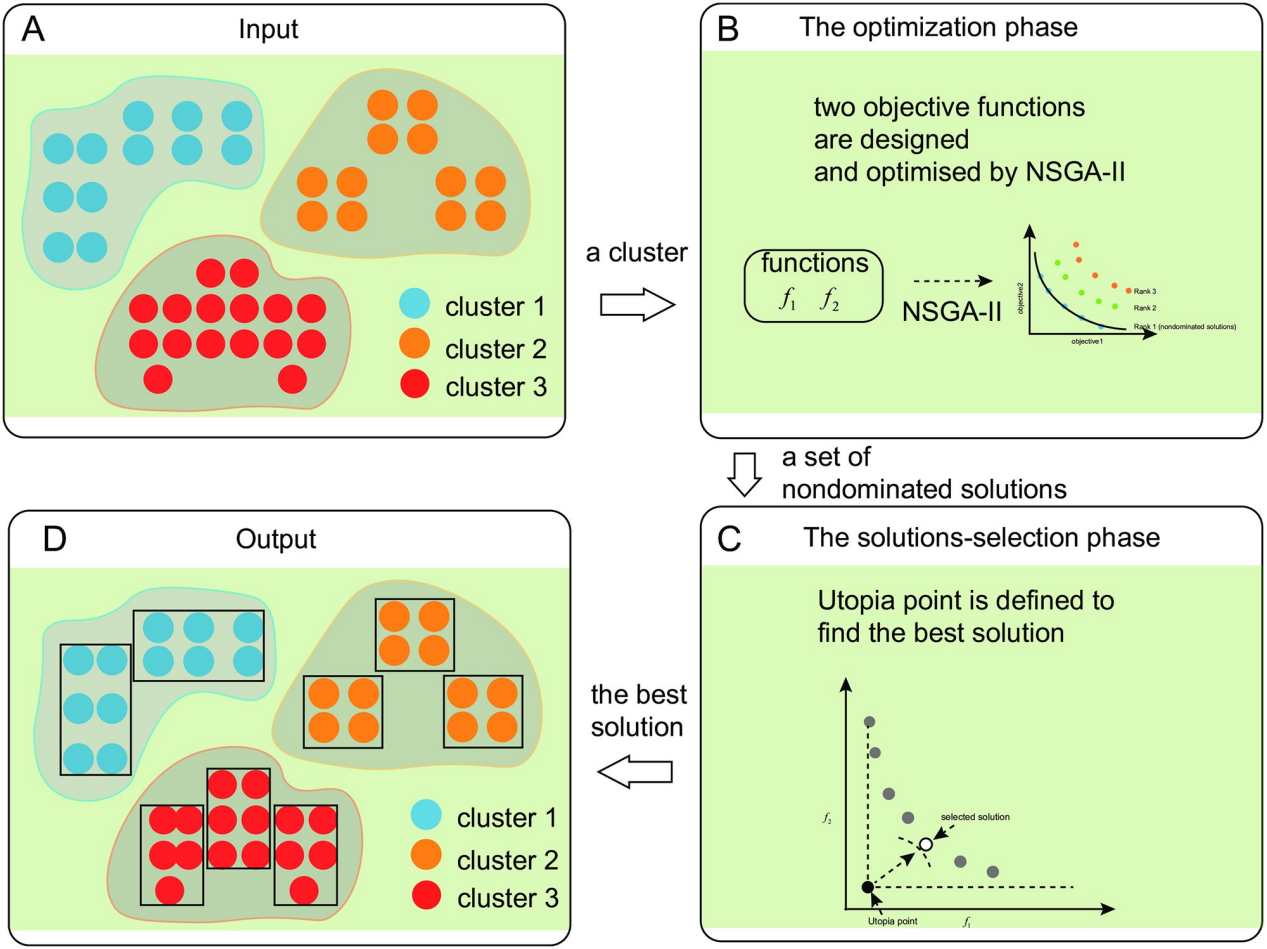
Overview of HcubeOM. (A) is the result of the traditional clustering method, where blue, red, and orange represent a cluster respectively. (B) is the optimization phase. (C) is the solutions-selection phase. (D) is the output of HcubeOM, where the black rectangles represent hypercubes for each cluster. Based on these hypercubes, the explanations of each cluster can be generated.

The proposed hypercube overlay model employs NSGA-II scheme and consists of two main phases: optimization and solution selection. In the first optimization phase, two objective functions are designed and optimized with NSGA-II, where simulated binary crossover and polynomial mutation are used to update population and medoid-based coding is used to generate individuals. The output of this first phase is a set of mutually nondominated solutions, which correspond to different tradeoffs between the two objectives. In the second solution selection phase, an Utopia point is defined to determine the most suitable solution from the set, in which each cluster can be covered by as few hypercubes as possible. The overview of HcubeOM is illustrated in Fig 3.

### Two objective functions

**Fig 4:**
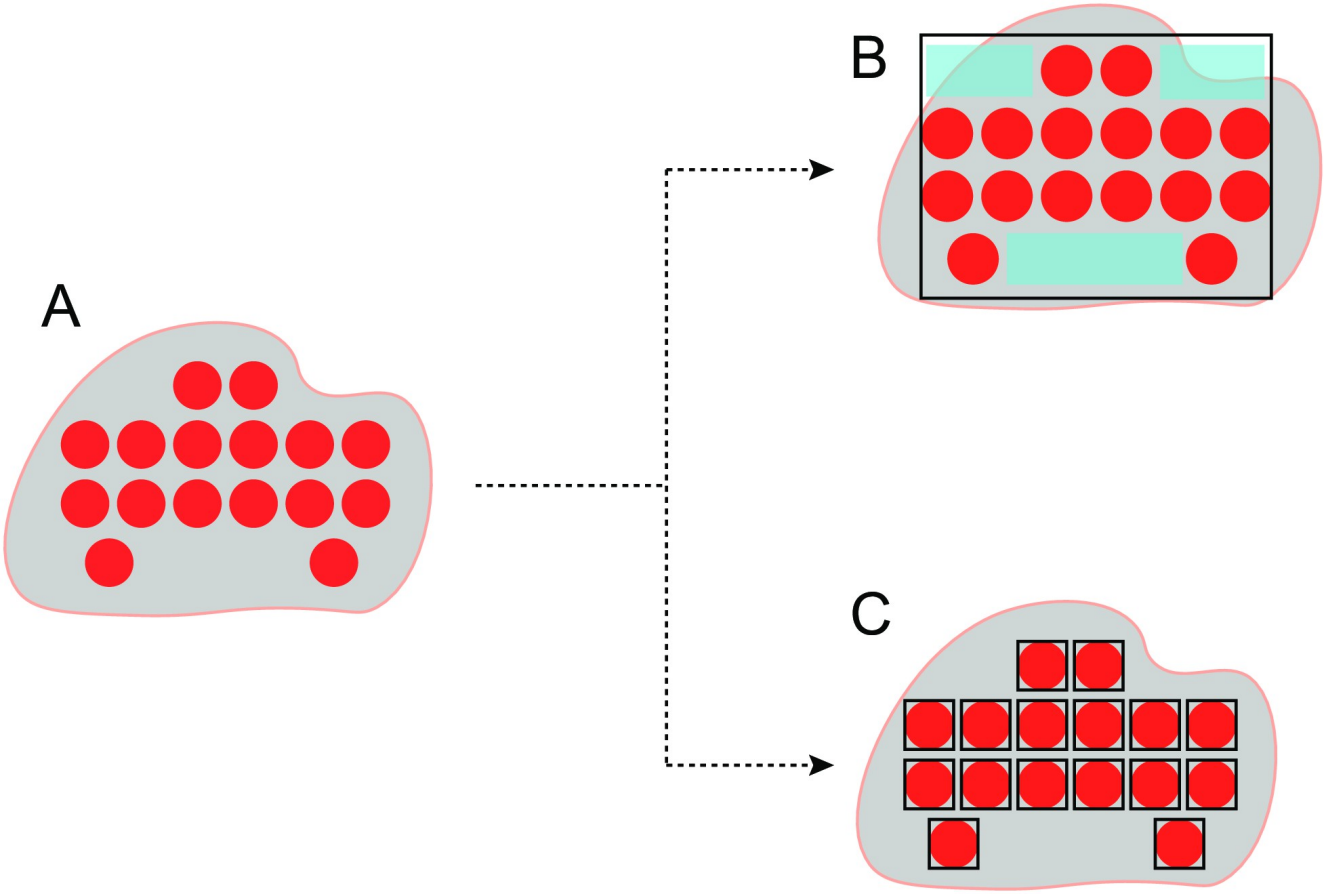
The result with different hypercubes. (A) is the result of a cluster. (B) is the result with only one hypercube, the explanations generated from the light blue part are meaningless and redundant. (C) is the result with multiple hypercubes, where each instance is deemed as a hypercube.

To explain a clustering result, good values of two indicators are normally expected to be achieved: succinctness and accuracy. Succinctness means that the explanation of a cluster involves as few rules as possible, while accuracy means those rules cover the objects in the cluster well, namely, less objects of the cluster are missed and less objects out of the cluster are covered. Specifically, when hypercubes are employed to overlay a cluster, the two indicators can be illustrated as in Fig 4.

Fig 4(A) is a cluster. In Fig 4(B), only one hypercbe is used to explain the cluster, which results in a simple explanation. But the accuracy of the explanation is not so good, since the hypercube covers more redundant regions, and as a result, the probability of including objects out of the cluster could be high. Different from the overlay scheme in Fig 4(B), that in Fig 4(C) goes to the other extreme, where each object is covered by a hypercube. Although there is not any redundant region, the number of hypercubes is large, and the explanation of the cluster is much complicated.

Based on the above observation, two objective functions are developed. The first function *f*_1_ is directly defined as

$$\begin{array}{c} f_{1}\left(C_{q}\right)=k_{q} \end{array}$$ (2)

where *k*_*q*_ is the number of hypercubes for overlaying cluster *C*_*q*_, *C*_*q*_ ∈ *C* and *C* is a clustering.

The second function *f*_2_ is to minimize the compactness of instances within the hypercubes, and is defined as

$$\begin{array}{c} \begin{array}{c} f_{2}\left(C_{q}\right)=\frac{1}{k_{q}}\sum_{i=1}^{k_{q}}\;(\frac{\prod_{j=1}^{d}l_{ij}}{N_{i}}) \end{array} \end{array}$$ (3)

where *N*_*i*_ is the number of instances in the *i*th hypercube, *d* is the dimension of the dataset, and *l*_*ij*_ is the length of the *i*th hypercube in the *j*th dimension.

Intuitively, *f*_2_ is the average compactness of all hypercubes in a cluster.

**Fig 5:**
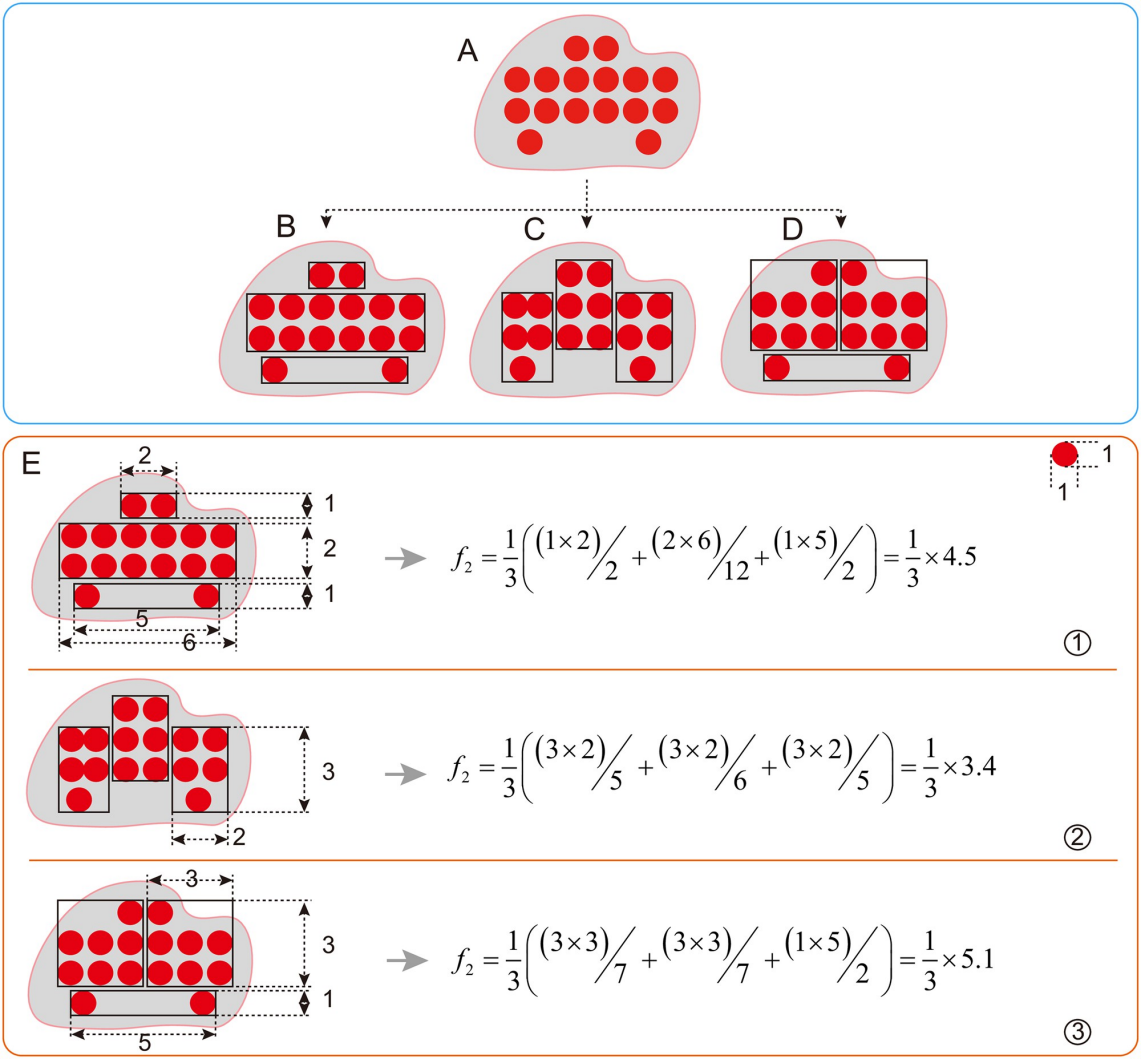
Quantification of compactness. (A) is a cluster. (B), (C) and (D) represent different overlay schemes respectively, of which each has three hypercubes. (E) illustrates the calculation process of the average compactness of the three overlay schemes.

For a specific number of hypercubes, there exist many different coverage schemes. For example, three different coverage schemes of a cluster in Fig 5 are presented and each with three hypercubes. Obviously, the scheme in Fig 5(C) is the best and that in (D) is the worst. According to function *f*_2_, the average compactness of the three schemes are calculated as in Fig 5(E), and the results do conform the observation.

When the number of hypercubes decreases, the compactness of instances will deteriorate. There is an opposing relationship between the two objective functions. Since it is necessary to find a solution with the smallest number of hypercubes, and the largest compactness of hypercubes, the final objective function of the multi-objective optimization is Eq 4.

$$\begin{array}{c} \begin{array}{c} Min\;F\left(C_{q}\right)=\{f_{1}=k_{q},f_{2}=\frac{1}{k_{q}}\sum_{i=1}^{k_{q}}\;(\frac{\prod_{j=1}^{d}l_{ij}}{N_{i}})\} \end{array} \end{array}$$ (4)

HcubeOM optimizes one result cluster at a time. Assuming *C*_*q*_ is the cluster to be optimized and all the following operations are illustrated in cluster *C*_*q*_.

### Initialization of solutions

Suppose that *POP* = {*G*_1_, *G*_2_, …, *G*_*i*_, …, *G*_*NP*_} is the population, the number of hypercubes for *C*_*q*_ is *k*_*q*_, *k*_*max*_ is the largest number of hypercubes to overlay a cluster, and it is discussed in the parameters section, where *G*_*i*_ is the *i*th individual, *NP* is the number of individuals, and each individual denotes a solution. The initial solutions can be generated by the following two steps.

Firstly, the values in interval [1..*k*_*max*_] are cyclically assigned to the individuals as their numbers of hypercubes so that the numbers cover all values from 1 to *k*_*max*_ and each will have the same frequency of occurrence. Suppose *k*_*i*_ ∈ {1, 2, …, *k*_*max*_} is the number of hypercubes of *i*th individual. *k*_*i*_ data points are randomly selected as medoids from the target cluster *C*_*q*_, and then each data point of *C*_*q*_ is assigned to the nearest medoid to generate *k*_*i*_ subcluster labels, as in Eq 5:

$$\begin{array}{c} \begin{array}{c} \underset{j}{\text{arg}\;\text{min}}{\left\|x-m_{j}\right\|}^{2},x\in C_{q},j\in \left\{1,2,3,...,k_{i}\right\}. \end{array} \end{array}$$ (5)

where *x* is a data point in *C*_*q*_, *m*_*j*_ is the *j*th medoid chosen in *C*_*q*_.

**Fig 6:**
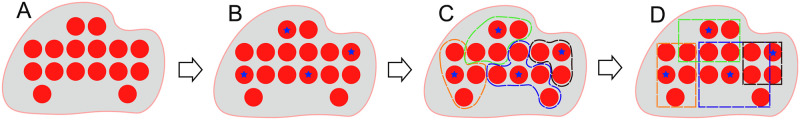
Initialization of the solutions. (A) is the target cluster *C*_*q*_. The data points with asterisks in (B) are the four randomly selected medoids. (C) is the result of four subclusters. (D) is the solution with 4 hypercubes for *C*_*q*_.

Secondly, the hypercubes of each subcluster are determined according to the maximum and minimum values of the subcluster in each dimension. An example of the initialization is shown in Fig 6.

### Solution encoding

The medoid-based individual encoding is employed to make the transformation among solutions, of which the idea is similar to that in [32].

**Fig 7:**
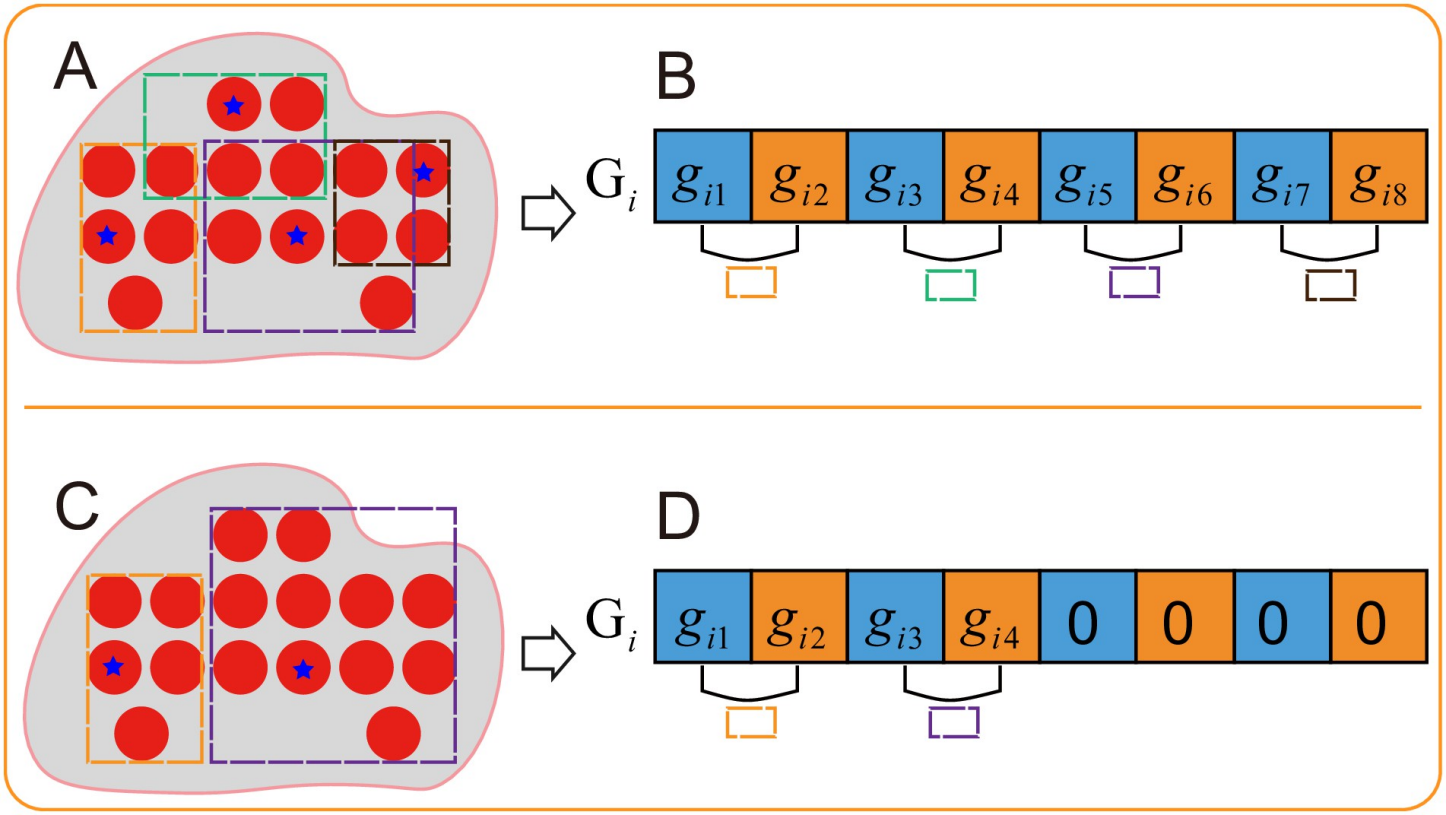
Solution encoding. *d* = 2, *k*_*max*_ = 4, *n* = 2 × 4 = 8, each two values is a group representing a hypercube. (A) is a solution with 4 hypercubes and (B) is the corresponding solution encoding. (C) is a solution with 2 hypercubes and (D) is the corresponding solution encoding.

Let *G*_*i*_ = {*g*_*i*1_, …, *g*_*id*_, *g*_*i*(*d*+1)_…, *g*_*i*(2*d*)_, *g*_*i*(2*d*+1)_…, *g*_*in*_} is the *i*th solution, where *n* is equal to (*d* × *k*_*max*_), *d* is the dimension of the dataset, and each group of *d* values represent the coordinate of a hypercube medoid. When the number of hypercubes *k*_*i*_ of a solution is less than *k*_*max*_, coordinates of the *k*_*i*_ medoids are assigned to the first (*d* × *k*_*i*_) values, and the rest values are set to 0. The representation of a solution is shown in Fig 7.

The advantage of this encoding scheme is that the representation is relatively short, since only the centriods are considered and the number of features are normally less than the number of the data points.

### Crossover and mutation

In the proposed method, simulated binary crossover and polynomial variation schemes are adopted to update the population, which have been extensively used in genetic-based clustering methods [33].

#### Simulated binary crossover

**Fig 8:**
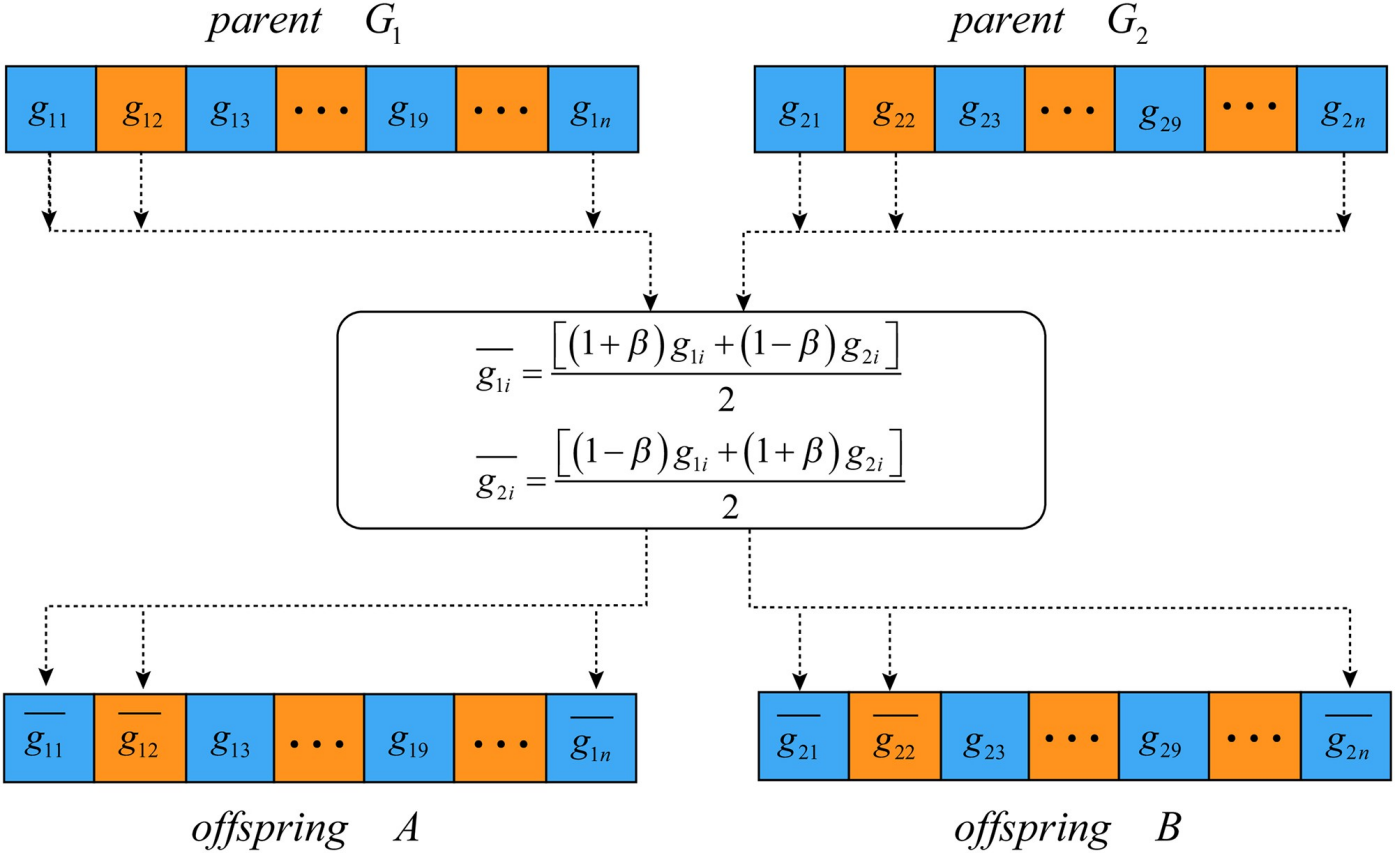
Crossover of two parent solutions. *parent* *G*_1_ and *parent* *G*_2_ are two selected parent solutions, *offspring A* and *offspring B* are the corresponding offspring. As crossover is not performed at from position 3 to 9, the genes of those positions are copied from the parents to the corresponding positions of the offspring. The other positions of the offspring will have new values.

Simulated binary crossover is one of the most popular crossover operators for continuous variables [34]. Consider that *G*_1_ = {*g*_11_, …, *g*_1*d*_, *g*_1(*d*+1)_…, *g*_1(2*d*)_, *g*_1(2*d*+1)_…, *g*_1*n*_} and *G*_2_ = {*g*_21_, …, *g*_2*d*_, *g*_2(*d*+1)_…, *g*_2(2*d*)_, *g*_2(2*d*+1)_…, *g*_2*n*_} are two selected parent solutions, where each group of *d* values represent the coordinate of a hypercube medoid, and *offspring A* and *offspring B* are generated by the crossover operation and the process is shown in Fig 8.

Two steps are included in the crossover operation. The first step is to determine which hypercube will to be changed, that is, which coordinators of hypercube centriods are selected for crossover. Particularly, a part of a coordinator can be divisively selected. For example, in *G*_1_, any position from *g*_11_ to *g*_1*n*_ can be selected solely. To keep the random property of the evolutionary, a random decimal fraction is generated for each position. If the decimal is less than a predefined probability threshold, say *Pc*, the corresponding position needs to be crossed.

The second step is to generate the new values in the offspring by crossover, and which are the medoid coordinates of the new hypercube. Suppose $\bar{g_{1i}}$ and $\bar{g_{2i}}$ are new values of offspring, and the crossover is performed by Eq 6.

$$\begin{array}{c} \begin{array}{c} \left\{ \begin{array}{c} \bar{g_{1i}}=\frac{\left[\left(1+\beta \right)g_{1i}+\left(1-\beta \right)g_{2i}\right]}{2}\\ \bar{g_{2i}}=\frac{\left[\left(1-\beta \right)g_{1i}+\left(1+\beta \right)g_{2i}\right]}{2} \end{array}\right. \end{array} \end{array}$$ (6)

where *β* is a polynomial probability distribution, and is calculated as in:

$$\begin{array}{c} \begin{array}{c} \beta =\{\begin{array}{cc} {\left(2\times \text{rand}\right)}^{1/(1+\eta_{c})}, & \;\text{if}\;\text{rand}\;\leq \frac{1}{2}\\ {\left(\frac{1}{2-2\times \text{rand}}\right)}^{1/(1+\eta_{c})}, & \;\text{otherwise}\; \end{array} \end{array} \end{array}$$ (7)

where *rand* is a random decimal fraction, and *η*_*c*_ is the distribution index. A larger *η*_*c*_ will lead to a offspring that is close to the parent solution, while a smaller *η*_*c*_ will result in a offspring that is significantly different from the parent solution.

#### Polynomial mutation

Polynomial mutation operation uses the polynomial probability distribution to perturb a solution to make a new solution [35].

**Fig 9:**
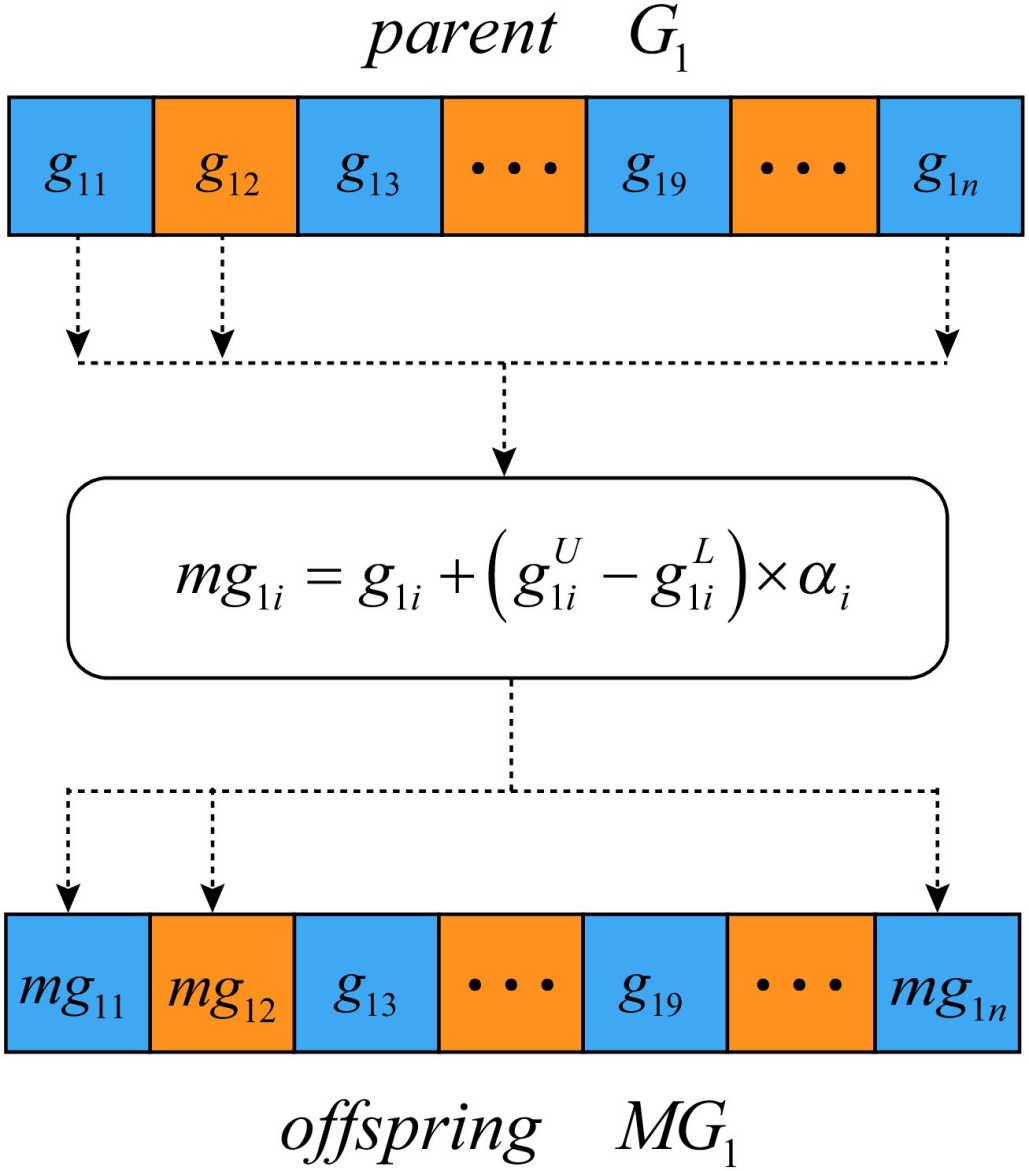
Mutation of parent solutions. *parent* *G*_1_ is the selected parent solution. *offspring* *MG*_1_ is the generated offspring. Mutation is not performed at positions 3 and 9. The value of *g*_13_ and *g*_19_ is directly copied to the positions of 3 and 9 of the offspring, and the other positions of offspring will have new values.

Suppose that *G*_1_ = {*g*_11_, …, *g*_1*d*_, *g*_1(*d*+1)_…, *g*_1(2*d*)_, *g*_1(2*d*+1)_…, *g*_1*n*_} is the selected parent, and the *offspring* *MG*_1_ is generated by the mutation operation and the process is shown in Fig 9.

The mutation operation is also composed of two steps. The first step is to determine which hypercube to be changed. For example, a random decimal fraction is generated for each position of *G*_1_, and if the decimal is less than a predefined probability threshold, say *Pm*, the corresponding position will be mutated.

The second step is to generate new values for the selected positions. Suppose *mg*_1*i*_ is new values of offspring *MG*_1_, and it is calculated by Eq 8.

$$\begin{array}{c} \begin{array}{c} mg_{1i}=g_{1i}+(g_{1i}^{U}-g_{1i}^{L})\times \alpha_{i} \end{array} \end{array}$$ (8)

where $g_{1i}^{U}$ and $g_{1i}^{L}$ are the upper and lower bounds of the decision variable *g*_1*i*_, and are set to the maximum and minimum values of the corresponding dimension, respectively. $g_{1i}^{U}-g_{1i}^{L}$ is the maximum value of perturbation allowed between parent and offspring, and *α*_*i*_ is the perturbation factor and is calculated as in:

$$\begin{array}{c} \begin{array}{c} \alpha_{i}=\{\begin{array}{cc} ({2\times \text{rand})}^{1/(1+\eta_{m})}-1, & \;\text{if}\;\text{rand}\;<\frac{1}{2}\\ 1-{\left(2-2\times \text{rand}\right)}^{1/(1+\eta_{m})}, & \;\text{if}\;\text{rand}\;\geq \frac{1}{2} \end{array} \end{array} \end{array}$$ (9)

where *rand* is a uniformly distributed random number in [0, 1], and *η*_*m*_ is the mutation distribution index.

#### Handling of out-of-bound value

**Fig 10:**
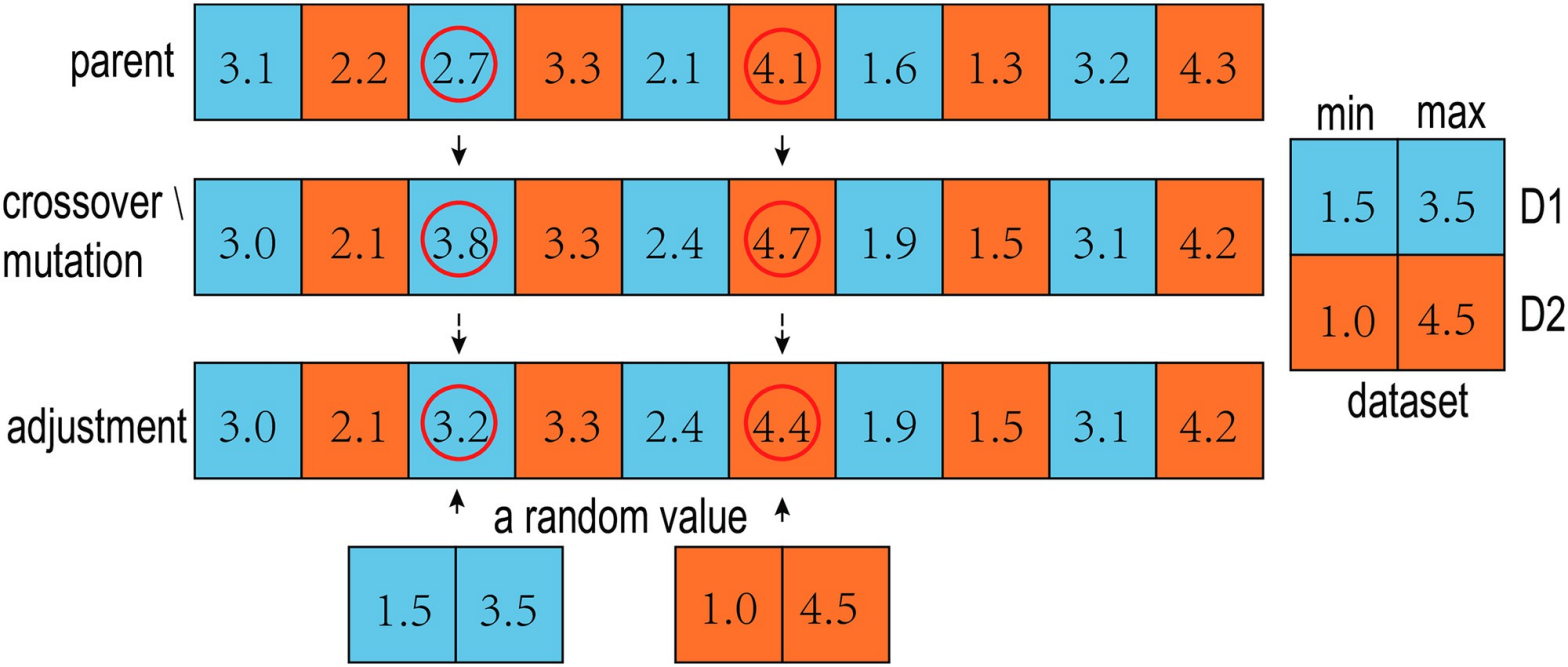
The adjustment process of the out-of-bound values. [1.5, 3.5] is the interval of dimension *D*_1_, and [1.0, 4.5] is the interval of dimension *D*_2_. In the third position of *parent* the value is 2.7, after crossover and mutation, it changed into 3.8, which is out of [1.5, 3.5]. During the adjustment, a random value, say 3.2, is taken from [1.5, 3.5] to replace 3.8. In the sixth position of *parent*, the value is 4.1, and becomes 4.7 after crossover and mutation operations. As 4.7 is not in [1.0, 4.5], a random value, say 4.4, is generated to replace 4.7.

After the crossover and mutation, one position value of the offspring may be not in the interval between the minimum and maximum values of the corresponding dimension. We call this position value out-of-bound. Obviously, this case is not reasonable, and we need to adjust those values out-of-bound. Fig 10 is an example of adjustment of the out-of-bound values. *parent* is the selected parent, *crossover*/*mutation* is the result of the crossover and mutation operation, and *adjustment* is the result of the adjustment. The values at position 3 and 6 in *crossover*/*mutation* are out-of-bound, and are replaced by random values within the intervals.

Combining crossover and mutation operations, the pseudocode of HcubeOM is presented in Algorithm 4.

In line 1, a population with 2*NP* is created. In line 2, [1, 2, 3, …, *k*_*max*_] array is created whose let the number of hypercubes in the population are not exactly the same. For each individual in the population the fitness is calculated from line 3 to 8. In line 9, all non-dominated fronts of the population are obtained using Algorithm 2, and the result is denoted by the set *F* = *F*_1_, *F*_2_, …, *F*_*i*_, …, where *F*_*i*_ is the set of all individuals in the *i*th non-dominated front, and *F*_1_ is the best level.

From line 10 to 12, crowding distances are calculated for all individuals in each non-dominated front using Algorithm 3. In line 13, individuals in the populations are sorted according to results of Algorithm 2 and Algorithm 3. In line 14, the top *NP* individuals in the sorted population are taken to form *P*_0_, which is the initial parent population.

From line 16 to 37 is the main loop of NSGA-II. In line 17, a child population is created by crossover and mutation operations presented in 1 and 1. In line 18, a combined population *R*_*t*_ = *P*_*t*_ ∪ *Q*_*t*_ is formed, and population *R*_*t*_ will be of size 2*NP*. From line 19 to 23, the fitness of each individual in *R*_*t*_ is calculated. Between line 24 and 27, all non-dominated fronts and crowding distances of *R*_*t*_ are calculated. From line 30 to 36, *NP* individuals in the sorted *R*_*t*_ are selected to form *P*_*t*+1_, which is the parent population for the next iteration.

**Algorithm 4** HcubeOM

**Input:** Maximum generation *maxGen*. Population size *NP*. Largest number of hypercubes *k*_*max*_. A cluster *C*_*q*_, *C*_*q*_ ∈ *C* and *C* is a clustering.

**Output:** a set of optimal solutions *F*_1_

1: Initialize 2*NP* solutions with *k*_*max*_ hypercubes, *POP* = {*G*_1_, *G*_2_, …, *G*_*i*_, …, *G*_2*NP*_}.

2: set nums = [1, 2, 3, …, *k*_*max*_]

3: **for** *G*_*i*_ ∈ *POP* **do**

4:  Assign nums[*i* mod *k_max_*] to *G*_*i*_

5:  Determine the decision variables for individual *G*_*i*_

6:  Find hypercubes of individual *G*_*i*_.

7:  Calculate the value of the *f*_1_ and *f*_2_ for individual *G*_*i*_.

8: **end for**

9: *F* = fast-nondominated-sort (*POP*).

10: **for** *F*_*i*_ ∈ *F* **do**

11:  crowding-distance (*F*_*i*_).

12: **end for**

13: Individuals in *POP* are sorted according to results of fast-nondominated-sort and crowding-distance

14: *P*_0_ = *POP*[1 : *NP*]

15: *t* = 0

16: **while** *t* ≤ *maxGen* **do**

17:  generate child population *Q*_*t*_ from *P*_*t*_ using crossover and mutation

18:  set *R*_*t*_ = *P*_*t*_ ∪ *Q*_*t*_

19:  **for** *G*_*i*_ ∈ *R*_*t*_ **do**

20:   Determine the decision variables for individual *G*_*i*_

21:   Find hypercubes of individual *G*_*i*_.

22:   Calculate the value of the *f*_1_ and *f*_2_ for individual *G*_*i*_.

23:  **end for**

24:  *F* = fast-nondominated-sort (*R*_*t*_)

25:  **for** *F*_*i*_ ∈ *F* **do**

26:   crowding-distance (*F*_*i*_)

27:  **end for**

28:  *P*_*t*+1_ = ∅

29:  *i* = 1

30:  **while** ∣*P*_*t*+1_∣ + ∣*F*_*i*_∣ < *NP* **do**

31:   *P*_*t*+1_ = *P*_*t*+1_ ∪ *F*_*i*_

32:   *i* = *i* + 1

33:  **end while**

34:  Sort *F*_*i*_ on crowding distance

35:  *P*_*t*+1_ = *P*_*t*+1_ ∪ *F*_*i*_[1 : (*NP* − ∣*P*_*t*+1_∣)]

36:  *t* = *t* + 1

37: **end while**

38: **return** *F*_1_

### Selection of the most suitable solution

**Fig 11:**
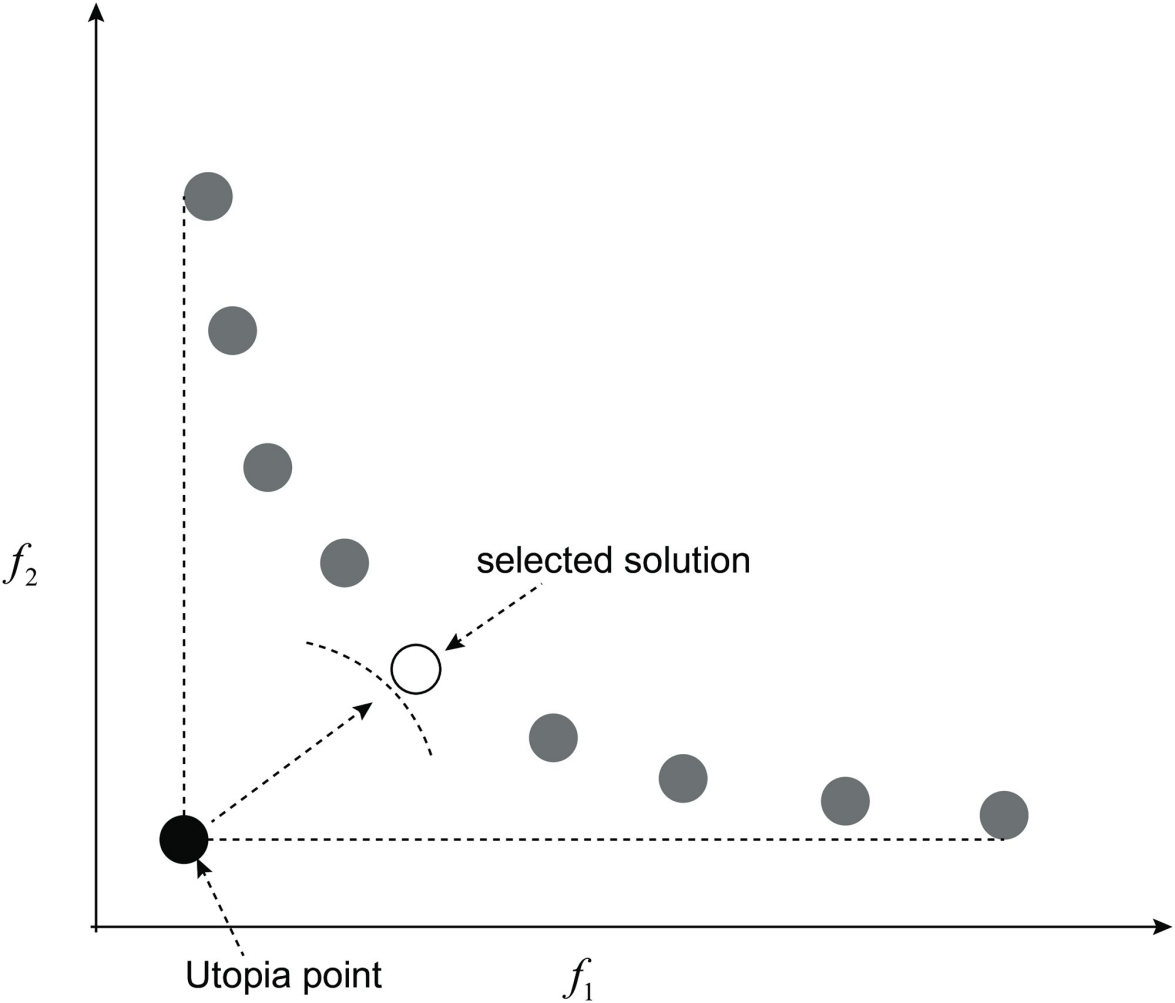
Selection of the most suitable solution. Distance technique to find final solution as the closest solution in the Pareto set to the Utopia location [36].

A distance-based technique is used to select the solution in the Pareto set with the minimum distance from an ideal solution, called Utopia point [36]. Utopia location is defined as the intersection point of the lines passing through the top left and bottom right solutions of the Pareto front in the area of possible outcomes, which is illustrated in Fig 11. In other words, the coordinate of the Utopia point is the best value obtained for the objective functions during the optimization process. The specific process is as shown in Algorithm 5.

**Algorithm 5** Selection of the most suitable solution

**Input:** a set of optimal solutions *F*_1_

**Outout:** a best solution *F*_*best*_

 1: Find solutions for the endpoints on both sides of the solution set of *F*_1_

 2: Determine Utopia point based on two endpoints

 3: Find the closest solution to Utpia point in *F*_1_, set *F*_*best*_

 4: **return** *F*_*best*_

### Generation of explanations

This subsection describes in detail how the final explanatory statements are generated. Based on the optimal individuals derived in the previous section the hypercubes of each cluster can be determined.

**Fig 12:**
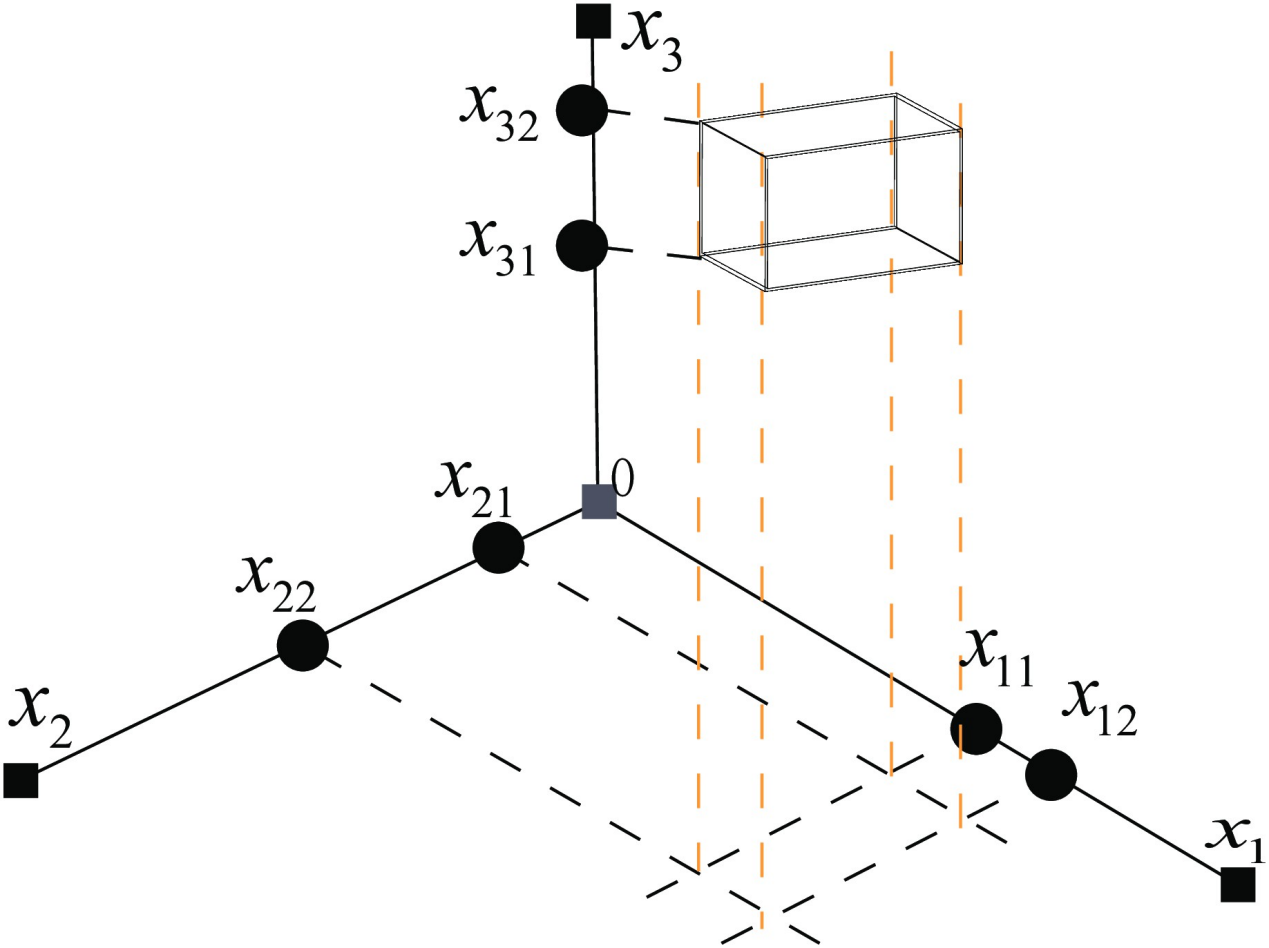
Generation of explanations. The black cube is a hypercube in one of the clusters, and *x*_11_, *x*_12_, *x*_21_, *x*_22_, *x*_31_, *x*_32_ are the endpoints obtained by mapping the hypercube to each feature dimension. *x*_11_ < *x*_12_, *x*_21_ < *x*_22_, *x*_31_ < *x*_32_.

Assuming that the black cube in Fig 12 is a hypercube of a certain cluster, the following will specify the generation process of the explanatory statements based on Fig 12. First, the hypercube is mapped to each feature dimension to obtain the endpoints of the hypercube’s edge lengths in each feature dimension, i.e. *x*_11_, *x*_12_, *x*_21_, *x*_22_, *x*_31_, *x*_32_ in Fig 12. Then the endpoints in each feature dimension are constructed into a one-dimensional array of one row and two columns, i.e., [*x*_11_, *x*_12_], [*x*_21_, *x*_22_], [*x*_31_, *x*_32_]. Finally, the one-dimensional array on all feature dimensions is constructed into a two-dimensional array with the number of rows equal to the number of features, i.e.[*x*_11_, *x*_12_; *x*_21_, *x*_22_; *x*_31_, *x*_32_]. The other hypercubes of the cluster are processed according to this procedure, and then the two-dimensional array obtained from all the hypercubes is stored in a list, which is called *ExSpace*. Enter *ExSpace* into Algorithm 6, which generates user-friendly explanatory statements.

In Algorithm 6, *ExSpace* serves as the input to the algorithm and *EndExplanation* is the output, which consists of user-friendly explanatory statements. Line 2 of the algorithm sets up a loop with a number of loops equal to the length of *ExSpace*, and each loop passes a two-dimensional array to the intermediate variable *cube*. Lines 4 through 8 implement the generation of explanatory statements. Line 5 is the loop with the number of loops equal to the number of features in the dataset, which is used to traverse the two endpoints identified on each feature dimension. Line 6 completes the string splicing with the strcat function to form the explanatory statement. At the end of the loop set in line 5, the explanatory statements generated by the currently traversed hypercube are stored in *src* in line 7. Line 10 uses *EndExplanation* to store the explanation statements for all hypercubes in the cluster, with the explanation statements between hypercubes connected by &.

For example, the two-dimensional array obtained from the hypercube in Fig 12 is [*x*_11_, *x*_12_;*x*_21_, *x*_22_;*x*_31_, *x*_32_]. When this two-dimensional array is traversed, it is passed to the intermediate variable *cube*. Line 5 then generates loops with a loop count of 3, each of which takes two endpoint values on a feature dimension from the *cube*, before generating an explanatory statement for that *cube* via the string concatenation function in line 6. After the first loop, the generated explanatory statement is [*x*_11_ ≤ *x*_1_ ≤ *x*_12_]. After the end of the loop set in the line 5 the final explanation statements for the hypercube will be available in *src*, i.e. *src* = [*x*_11_ ≤ *x*_1_ ≤ *x*_12_, *x*_21_ ≤ *x*_2_ ≤ *x*_22_, *x*_31_ ≤ *x*_3_ ≤ *x*_32_]. When the loop set in line 2 is finished, all explanatory statements for the input cluster are available in *EndExplanation*.

**Algorithm 6** Generation of explanations

**Input:** *ExSpace*

**Output:** *EndExplanation*

1: *endSrc* = [ ]

2: **for** *j* = 1, 2, …, size(*ExSpace*, 1) **do**

3:  *cube* = *ExSpace*(j).

4:  *src* = [ ]

5:  **for** *i* = 1, 2, …, size(*cube*, 1) **do**

6:   *str* = strcat(int2str(*cube*(i,1)),≤,*x*_*i*_,≤,int2str(*cube*(i,2))).

7:   *src* = [*src*, *str*]

8:  **end for**

9:  *EndExplanation* = [endSrc, src, &]

10: **end for**

11: **return** *EndExplanation*

### Evaluation metrics

Two metrics are proposed to evaluate the performance of the obtained explanations in two aspects, namely the succinctness and consistency of explanations.

#### The evaluation metric of succinctness

The succinctness rate (SR) is defined to evaluate the explanation succinctness of each cluster in two factors. The first factor is the number of hypercubes in a cluster, as the fewer the number, the simpler the explanation. The second factor is the portion of the redundant region in a hypercube, as the less the portion, the more accurate the explanation. Bear this intuition in mind, one can design the index SR as in:

$$\begin{array}{c} \begin{array}{c} SR=\frac{\sum_{q=1}^{C}(k_{q}\times \sum_{i=1}^{k_{q}}\;(\frac{\Pi_{j=1}^{d}l_{ij}}{N_{i}}))}{C} \end{array} \end{array}$$ (10)

where *C* is the number of clusters, *k*_*q*_ is the number of hypercubes in the *q*th cluster, *N*_*i*_ is the number of instances in the *i*th hypercube, *d* is the dimension of the dataset, and *l*_*ij*_ is the length of the *i*th hypercube in the *j*th dimension. *k*_*q*_ is the very first factor, and $\sum_{i=1}^{k_{q}}(\prod_{j=1}^{d}l_{ij}/N_{i})$ is the average hyper-volume of each data point, which is used to indirectly and roughly depict the amount of redundant region. The smaller the SR, the better the succinctness of the resulting explanation.

However, some existing methods of clustering explanation are based on decision tree, and in which no concept of hypercube is involved. As a result, the number of hypercubes *k*_*q*_ cannot be calculated directly. But one can instead use closed intervals in the dimensions to act as hypercubes. The concrete process is described as follows.

**Fig 13:**
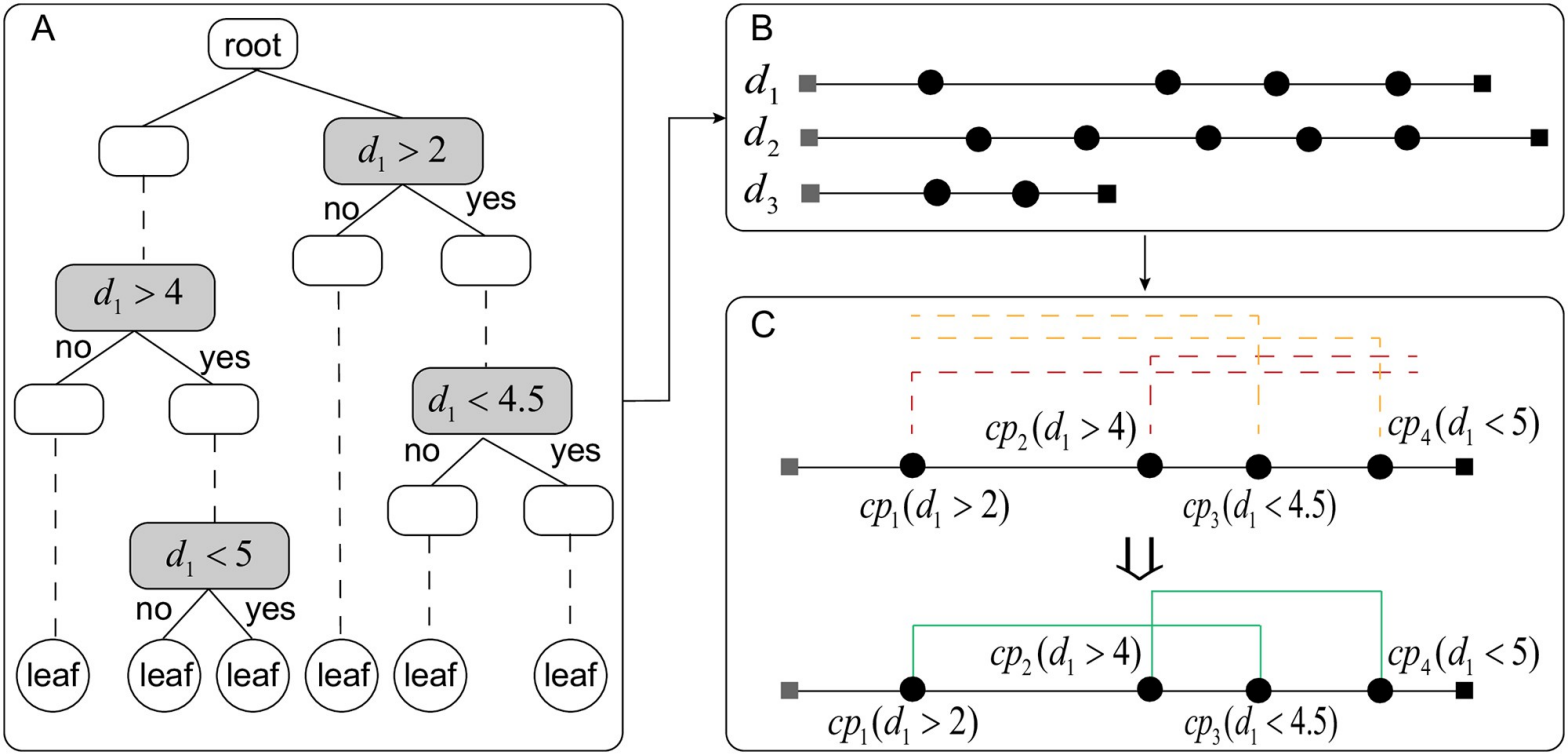
Decision tree nodes transformed into closed intervals. (A) is a decision tree, in which an ellipse denotes a cut point, a circle denotes a leaf node, and the gray ellipses are the cut points on *d*_1_, and the dashed lines indicate the cut points are omitted. (B) is the distribution of cut points in *d*_1_, *d*_2_ and *d*_3_ dimension. The black circles are cut points and the squares are the endpoints. (C) is the process of determining the closed interval on *d*_1_ dimension. The dashed line are the cutting rules and the solid line are the closed intervals obtained.

In Fig 13, the closed intervals on a dimension is shown. Suppose that the decision tree has 4 cut points in dimension *d*_1_, which are *cp*(*d*_1_ > 2), *cp*_2_(*d* > 4), *cp*_3_(*d*_1_ < 4.5) and *cp*_4_(*d*_1_ < 5). The four cut points form two closed intervals, namely from 2 to 4.5 and from 4 to 5, in terms of the following rule: Each cut point with greater than operator, from the leftmost to the rightmost along a dimension, combines with its nearest neighbor with less than operator to generate a closed interval. The intuition behind this is that one can avoid the case that an interval might enclose another. For example, if *cp*_1_(*d*_1_ > 2) and *cp*_4_(*d*_1_ < 5) is an interval, and *cp*_2_(*d*_1_ > 4) and *cp*_3_(*d*_1_ < 4.5) is the other one, then the former encloses the later. This will lead to a bad explanation. If the total number of cut points is odd, the last one will form a closed interval together with the maximum value of this dimension.

**Fig 14:**
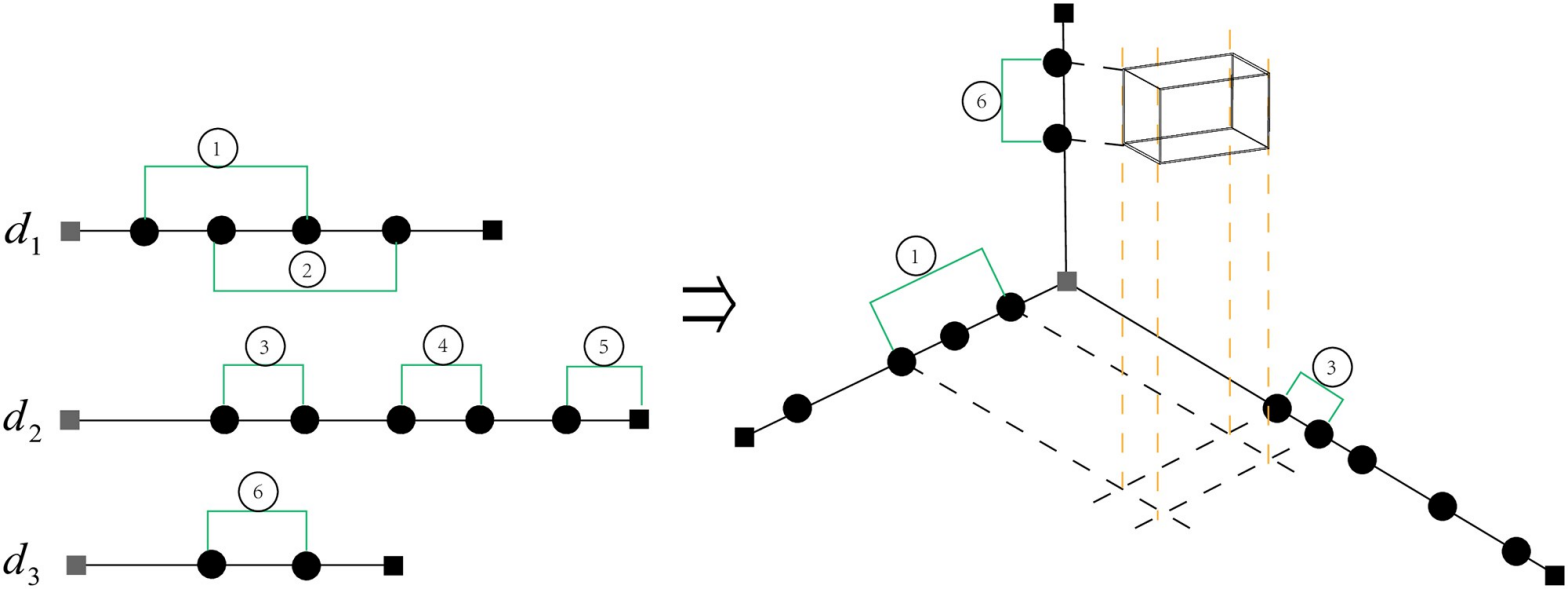
Composition of hypercubes from closed intervals. The process of determining the hypercube from the closed interval. The left is the six closed intervals in the three dimensions, while the right is the cube determined by [① ③ ⑥] intervals.

Fig 14 illustrates the closed intervals from different dimensions form a hypercube. For example, interval ①, ③ and ⑥ are from *d*_1_, *d*_2_ and *d*_3_, respectively, and they constitute a hypercube. In this way, the closed intervals will generate 6 candidate hypercubes, which are [① ③ ⑥],[① ④ ⑥],[① ⑤ ⑥], [② ③ ⑥], [② ④ ⑥], [② ⑤ ⑥]. However, the hypercubes without data objects inside will be removed, and the remaining ones are the valid hypercubes that can be used for metric calculation.

#### The evaluation metrics of consistency

Although one expects the explanation of a clustering can describe the clustering as accurate as possible, some gaps might exist. Therefore, we can measure the consistency between explanation and clustering to evaluate the explanation quality. Two metrics are used herein to quantify this consistency.

The first metric is Error Rate (ER) [37], which quantifies consistency from the perspective of wrong classification as follows.

$$\begin{array}{c} \begin{array}{c} ER=[(\sum_{m=1}^{S-1}\sum_{n=m+1}^{S}|A_{mn}-B_{mn}|)/(\frac{S(S-1)}{2})] \end{array} \end{array}$$ (11)

where *S* is the size of instances. If instances *x*_*m*_ and *x*_*n*_ are the members of the same cluster in the clustering, then *A*_*mn*_ is 1, otherwise, *A*_*mn*_ is 0. If instances *x*_*m*_ and *x*_*n*_ are still the members of the same cluster in the explanation to be measured, then *B*_*mn*_ is 1, otherwise, *B*_*mn*_ is 0. ER is the error rate of the consistency, and the lower the value, the more matched the explanations with the clustering.

Another metic is True Positive Rate (TPR) [38], which quantifies consistency from the perspective of right classification as follows.

$$\begin{array}{c} \begin{array}{c} \text{TPR}=\frac{1}{S}\sum_{q=1}^{C}(\sum_{x_{a},x_{b}\in C_{q}}\text{agree}(x_{a},x_{b})) \end{array} \end{array}$$ (12)

where *S* is the size of instances, *C* is the number of cluster, *C*_*q*_ is the *q*th cluster *x*_*a*_ and *x*_*b*_ are the specific instances in the *q*th cluster. If *x*_*a*_ and *x*_*b*_ are still in the same cluster in the explanation, then *agree*(*x*_*a*_, *x*_*b*_) is 1, otherwise, it is 0. The larger the value of TPR, the more consistency between the explanations and the clustering. Based on these two metrics, it can be determined whether the explanation can effectively reflect the clustering or not.

## Experimental result

### The methods of comparison

In this section, experiments are carried out to test HcubeOM and compare it with other four existing methods. The values of foregoing evaluation metrics are calculated with each method. Then, based on which, a comparative analysis is conducted among HcubeOM and other four methods in terms of consistency and succinctness of explanations.

ExKMC [39] has made some improvements to the decision tree, where the main contribution is the introduction of a parameter *k*′. In this method, a threshold tree with *k* leaf nodes is constructed, and then the *k* nodes are expanded to *k*′ nodes through greedy search. In this process, it is necessary to ensure that the proposed clustering cost has the smallest loss. In the end, a threshold tree with *k*′ nodes will be obtained, and each node of the tree can be used as the final explanations.

CUBT [40] uses the binary tree to achieve the explanations of clustering. This method consists of three stages. In the first stage, recursive partitioning algorithm is used to generate a tree with maximum depth. In the second stage, the tree is pruned based on the proposed minimum difference standard herein. In the final stage, the leaf nodes which can share the same node are merged. As binary partitioning of original variables is performed in the binary tree, this will help users understand the clustering result.

DReaM [27] explains clusters by establishing rules. One of its advantages is that two types of features can be specified randomly, the features of rule generation and the features of cluster structure preservation. In DReaM model, the features of rule generation are used to generate explanations for clustering, while those of cluster structure preservation are used for identifying clustering structure. Furthermore, the model also allows combination of priori knowledge to adjust the final rectangular decision boundaries.

CART [41] is a standard decision tree classification method. After the raw dataset is labeled through clustering, it is classified by CART. The nodes in the CART serve as the explanations for the cluster.

### Datasets and parameters

#### Datasets

Real life datasets Iris, Wine, BreastCancer, NewThyroid, and LiverDisorders are collected from the UCI Machine Learning Repository [42]. Adult is collected from [43]. *AD*_*_* is artificial dataset. *AD*_5_2 is collected from [44]. *AD*_4_2 is collected from [45]. *AD*_3_2, *AD*_3_4, and *AD*_3_6 are generated with sklearn.datasets.make_blobs method [46], they all take default parameters. The brief description of datasets is reported in Table 1 and they are available in https://github.com/cLiang113/dataset, where *F* is the number of features, *C* is the number of clusters, *S* is sample size.

**Table 1:** Summary of the datasets.

Dataset	F	C	S
AD_3_2	2	3	200
AD_3_4	4	3	200
AD_3_6	6	3	200
AD_4_2	2	4	200
AD_5_2	2	5	200
Iris	4	3	150
Adult	15	2	850
Wine	13	3	178
BreastCancer	10	2	699
NewThyroid	5	3	215
LiverDisorders	6	2	345

The description of the dataset is give below.

- *AD*_3_2: This dataset consists of 3 separated clusters with equal size.
- *AD*_3_4: This dataset contains 200 data point with 4 attributes distributed over 3 clusters.
- *AD*_3_6: This dataset has 3 clusters with unequal size.
- *AD*_4_2: This dataset contains 200 data points with 2 attributes distributed over 4 overlapping square-shaped clusters. It is generated by normal distributions with a standard deviation of two in both dimensions.
- *AD*_5_2: This dataset contains highly overlapped 200 data point with 2 attributes distributed over 5 clusters.
- Iris: This dataset composes of 3 clusters, where each cluster consists of 50 instances with four features. Each instance is described by four physical features which are length and width of sepals and petals.
- Adult: This dataset is changed by [43], where only “age” attribute and “pay” label are used in the experiment.
- Wine: This dataset consists of 178 instances with thirteen chemical attributes. These attributes are found in the chemical process of three different wines in the same region in Italy, but the data are derived from three different cultivars.
- BreastCancer: This dataset consists of 699 instances with 10 attributes. It originated from the Wisconsin breast cancer database, each instance is categorized within 2 clusters: benign or malignant, and these 2 clusters are linearly separated.
- Newthyroid: This dataset contains 3 clusters with 5 attributes of 215 data points. These attributes can identify whether patient’s thyroid gland functions are in normal condition, hypothyroidism or hyperthyroidism.
- LiverDisorders: This dataset contains two clusters with six attributes of 341 data points. This data is about whether a person suffers from alcoholism.

*F* is the number of features, *C* is the number of clusters, and *S* is the size of the dataset.

#### Parameters

The setting of parameters is shown in Table 2. Sklearn’s GridSearchCV module [46] is used to determine the values of each parameter in the algorithm. In GridSearchCV module, the search interval of population size *NP* is set to [50, 200] with a step size of 50. Maximum generation *maxGen* has a search space of [100, 400] with a step size of 100. Probability of crossover and mutation are set to [0, 1] with a step size of 0.2. *η*_*c*_, *η*_*m*_ and *β*, refered to [33], are set to [2, 10] with a step size of 1. The *k*_*max*_ parameter is the maximum number of hypercubes, which is similar to the maximum number of clusters parameter when solving clustering problems with multi-objective optimization, so *k*_*max*_ is set to $\left\lceil \sqrt{S}\right\rceil$ based on existing work on multi-objective clustering, ⌈ ⌉ is an upward rounding symbol that lets *k*_*max*_ be an integer.

**Table 2:** Setting of parameters.

Parameter	Setting
population size *NP*	100
Maximum generation *maxGen*	200
Probability of crossover	0.8
Probability of mutation	0.2
*η*_*c*_	5.0
*η*_*m*_	5.0
*β*	2.0
*k*_*max*_	$\left\lceil \sqrt{S}\right\rceil$ , S is sample size

### The example of explanations

**Fig 15:**
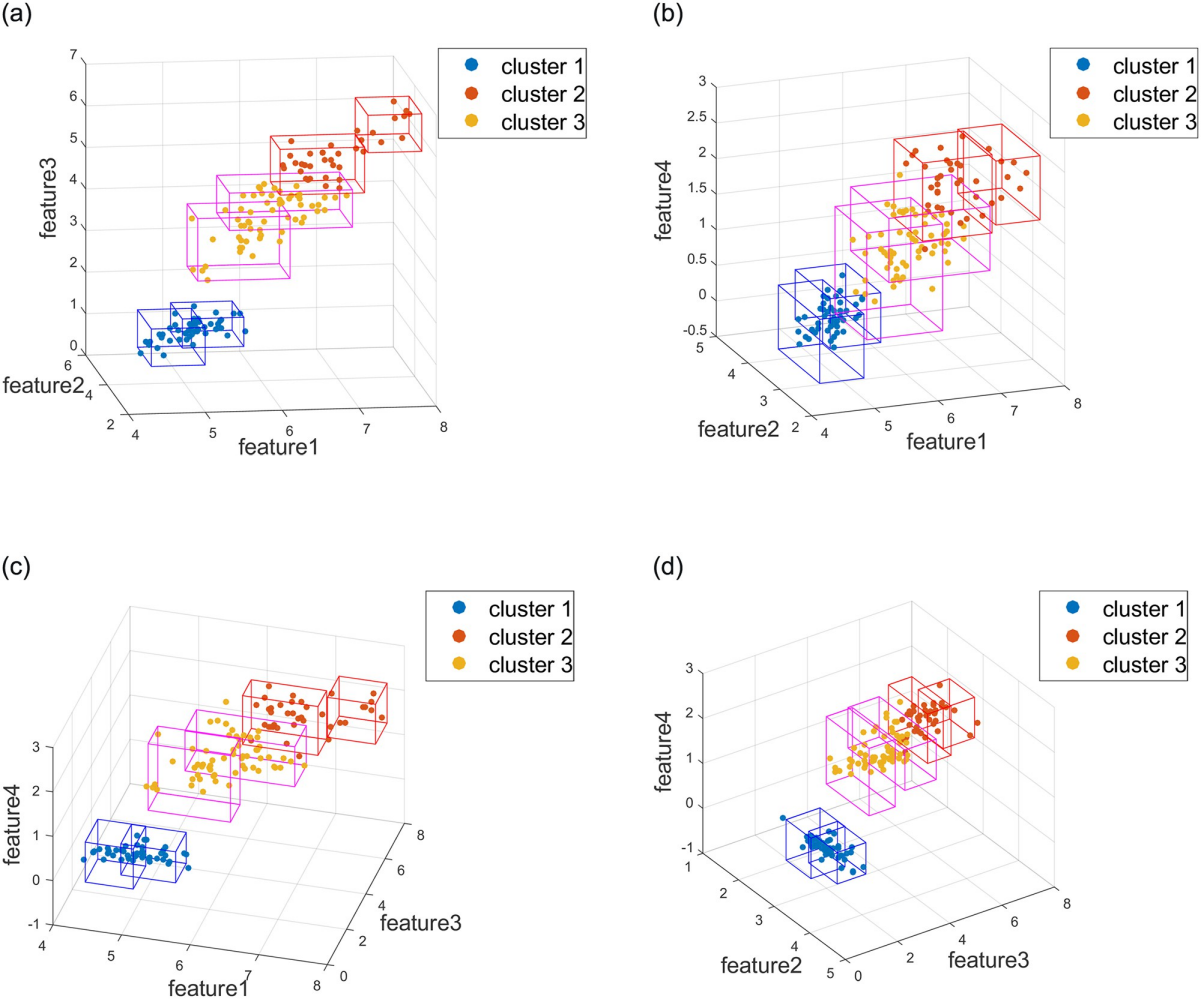
Hypercubes of each cluster in the Iris dataset obtained with HcubeOM. (a) Feature 1, Feature 2 and Feature 3. (b) Feature 1, Feature 2 and Feature 4. (c) Feature 1, Feature 3 and Feature 4. (d) Feature 2, Feature 3 and Feature 4.

**Fig 16:**
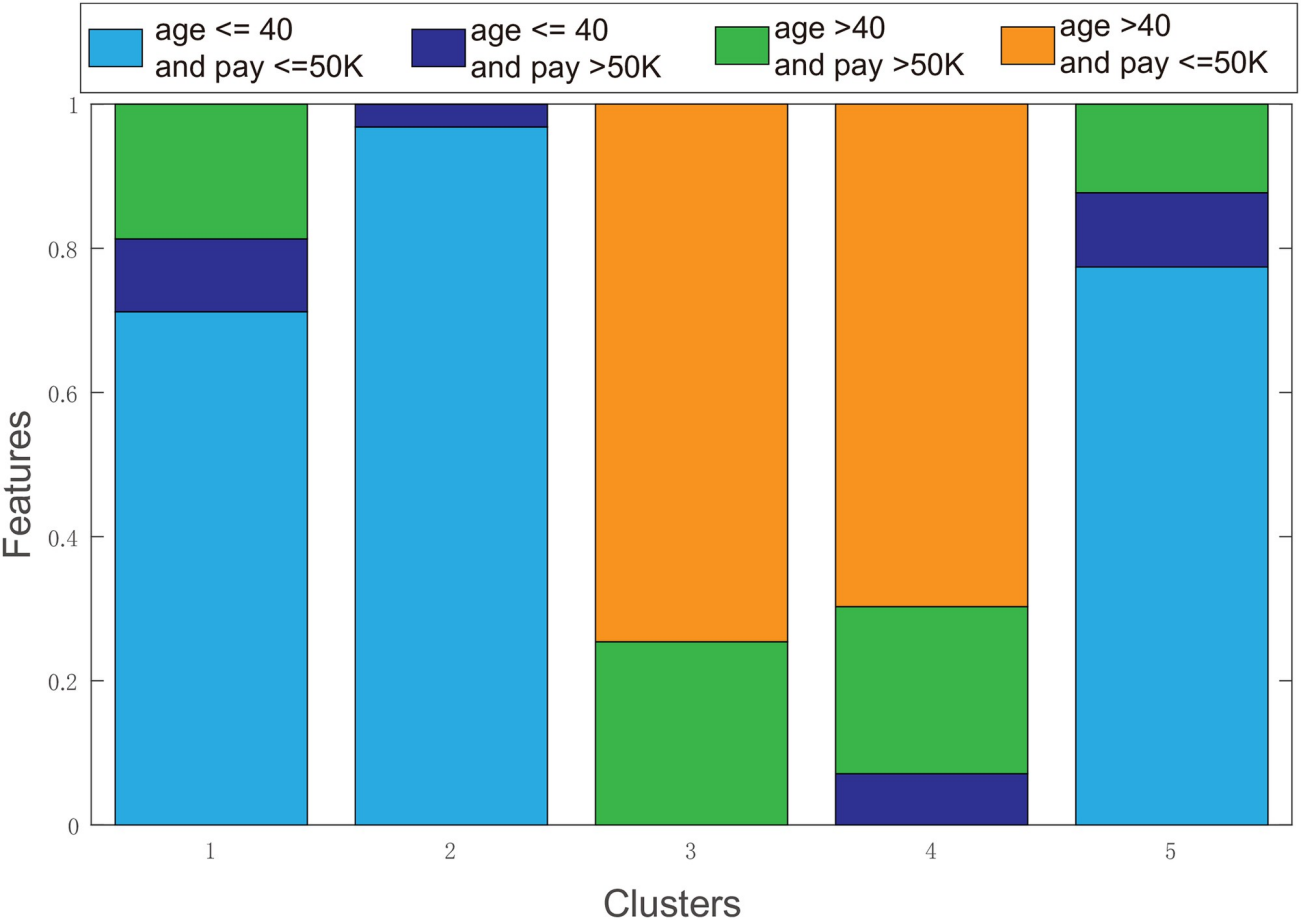
Distribution of features. The distribution of the two features of “age” and “pay” in Adult dataset.

Hypercubes obtained by HcubeOM can find regular decision boundarys for each cluster, which are combined to generate the explanations of each cluster. In this section, the experimental results of the Iris and Adult datasets are used to show the generation of explanations. Fig 15 shows the hypercubes for each cluster in the Iris dataset. Table 3 is the explanations of each cluster generated by the proposed method. Only two features, “age” and “pay”, are selected as explanatory features for the Adult dataset, which facilitates the display of explanatory results. The explanations for each cluster obtained with the proposed method is shown in Table 4. At the same time, the frequent pattern mining is used to obtain the ratio of the two features in each cluster. The results are shown in Fig 16. From the comparison between the diagrams and tables, it can be clearly seen that the explanations obtained with the proposed method is basically the same as the distribution of the two features in each cluster shown in the figure, which indicates that the proposed method can well explain each cluster.

**Table 3:** Explanations for each cluster in Iris dataset.

Clusters	Rule
Cluster 1	[5.0 ≤ *x*_1_ ≤ 5.8, 3.3 ≤ *x*_2_ ≤ 4.4, 1.2 ≤ *x*_3_ ≤ 1.9, 0.1 ≤ *x*_4_ ≤ 0.6] ∪ [4.3 ≤ *x*_1_ ≤ 5.0, 2.3 ≤ *x*_2_ ≤ 3.6, 1.0 ≤ *x*_3_ ≤ 1.9, 0.1 ≤ *x*_4_ ≤ 0.3]
Cluster 2	[7.2 ≤ *x*_1_ ≤ 7.9, 2.6 ≤ *x*_2_ ≤ 3.8, 6.0 ≤ *x*_3_ ≤ 6.9, 1.8 ≤ *x*_4_ ≤ 2.5] ∪ [6.1 ≤ *x*_1_ ≤ 7.2, 2.5 ≤ *x*_2_ ≤ 3.4, 4.9 ≤ *x*_3_ ≤ 6.0, 1.4 ≤ *x*_4_ ≤ 2.5]
Cluster 3	[4.9 ≤ *x*_1_ ≤ 6.1, 2.0 ≤ *x*_2_ ≤ 3.0, 3.0 ≤ *x*_3_ ≤ 4.5, 1.0 ≤ *x*_4_ ≤ 1.7] ∪ [5.4 ≤ *x*_1_ ≤ 7.0, 2.2 ≤ *x*_2_ ≤ 3.4, 4.2 ≤ *x*_3_ ≤ 5.1, 1.2 ≤ *x*_4_ ≤ 2.4]

**Table 4:** Explanations for each cluster in Adult dataset.

Clusters	Rule
Cluster1	age ≤ 40 and pay ≤ 50K & age ≤ 40 and pay >50K
Cluster2	age ≤ 40 and pay ≤ 50K
Cluster3	age >40 and pay ≤ 50K & age >40 and pay >50K
Cluster4	age >40 and pay ≤ 50K & age >40 and pay >50K & age ≤ 40 and pay >50K
Cluster5	age ≤ 40 and pay ≤ 50K & age ≤ 40 and pay >50K & age >40 and pay >50K

### The succinctness of explanations

In this section, the SR of each method is calculated based on the proposed succinctness metric. In order to reflect the robustness of the experimental results, each method is run 15 times on each dataset, and finally the mean and standard deviation of the 15 results are computed as the final comparison. When calculating SR, each feature dimension of the dataset is normalized so that each feature value of the dataset is in [0, 1]. The normalization prevents some eigenvalues of the dataset from being too large and affecting the presentation of the final SR results. The specific values of SR are shown in Table 5, the smaller the SR, the better the succinctness of explanations.

**Table 5:** Average SR values on different methods are compared, in which those in brackets are the corresponding standard deviations.

Dataset	HcubeOM	ExKMC	CUBT	CART	DReaM
AD32	3.7367E-03 (3.1563E-03)	4.0239E-03 (1.8868E-04)	4.0579E-03 (1.8692E-04)	4.1075E-03 (1.9279E-04)	**3.5101E-03** (1.8721E-04)
AD34	**4.7290E-04** (1.9459E-05)	5.0011E-04 (1.7259E-05)	5.7411E-04 (1.9908E-05)	5.8361E-04 (1.9592E-05)	5.3236E-04 (2.3453E-05)
AD36	**2.8950E-04** (2.0609E-06)	3.1616E-04 (1.9876E-06)	3.2716E-04 (2.2090E-05)	3.4069E-04 (2.6310E-05)	3.5011E-04 (2.3113E-06)
AD42	8.6033E-03 (2.3175E-03)	8.9205E-03 (2.7175E-04)	9.5045E-03 (3.0974E-04)	9.6554E-03 (3.1458E-04)	**8.3325E-03** (2.9865E-04)
AD52	1.0967E-02 (5.9843E-04)	1.1255E-02 (5.7928E-04)	1.9269E-02 (6.2660E-04)	2.1187E-02 (7.4764E-04)	**1.0439E-02** (3.1733E-04)
Iris	**1.5668E-03** (1.4484E-04)	1.8540E-03 (1.8984E-04)	2.1680E-03 (3.3289E-04)	2.3167E-03 (3.0770E-04)	1.9717E-03 (2.8784E-04)
Wine	**3.5608E-04** (8.3273E-06)	3.7134E-04 (3.0935E-05)	5.4534E-04 (3.6146E-05)	5.8516E-04 (3.2032E-05)	6.1289E-04 (9.6810E-06)
BreastCancer	**8.6468E-04** (2.8775E-05)	8.9319E-04 (2.8985E-05)	1.1659E-03 (2.9278E-05)	1.1444E-03 (2.9294E-05)	1.4346E-03 (2.9375E-05)
NewThyroid	**2.3070E-04** (5.8642E-05)	3.1790E-04 (6.9764E-05)	5.3190E-04 (7.4965E-04)	5.5787E-04 (7.1069E-04)	5.0034E-04 (7.3281E-05)
LiverDisorders	**3.2677E-03** (5.3125E-04)	3.5549E-03 (6.3766E-04)	4.5689E-03 (8.0163E-04)	4.1355E-03 (7.9833E-04)	4.8543E-03 (7.4665E-04)

It can be seen from the table that HcubeOM has good SR values on all datasets, which indicates the explanations obtained are relatively succinct and user-friendly. CUBT and CART all have poor value of SR. CART because itself is a decision tree. CUBT because a tree with maximum depth is formed during recursive partitioning of datasets in the first stage, each cluster will have a complex explanation generated based on the nodes of tree.

In dataset with low dimensions and relatively compact distribution. HcubeOM, ExKMC, and DReaM have very little difference. However, in datasets with higher dimensions and relatively dispersed distribution, HcubeOM is significantly better than the rest methods, mainly because the partitioning of clusters in DReaM is primarily done by a rectangular decision, so the rectangular decision will contain too many redundant parts for complex dataset. The explanations generated based on these parts are meaningless to the raw dataset, which brings some negative effects to the succinctness of explanations. The ExKMC method is a process of expanding the *k* nodes of tree to *k*′ nodes, where redundant nodes will be generated in some clusters for complex datasets. Therefore, ExKMC and DReaM are worse than HcubeOM in terms of succinctness of explanations for complex datasets.

The difference between the standard deviation of the different methods for each dataset is very small, indicating that all methods have similar and good robustness. In the datasets AD32, AD36, Iris, Wine, BreastCancer,NewThyroid,LiverDisorders, HcubeOM not only has good SR values, but also has the lowest standard deviation of the experimental results. But, in AD42,AD52,AD32, the standard deviation of HcubeOM is worse than some of the methods. This is mainly due to the fact that in low-dimensional datasets, there is a greater chance that the data point cluster labels will change when the decision boundary changes.

**Fig 17:**
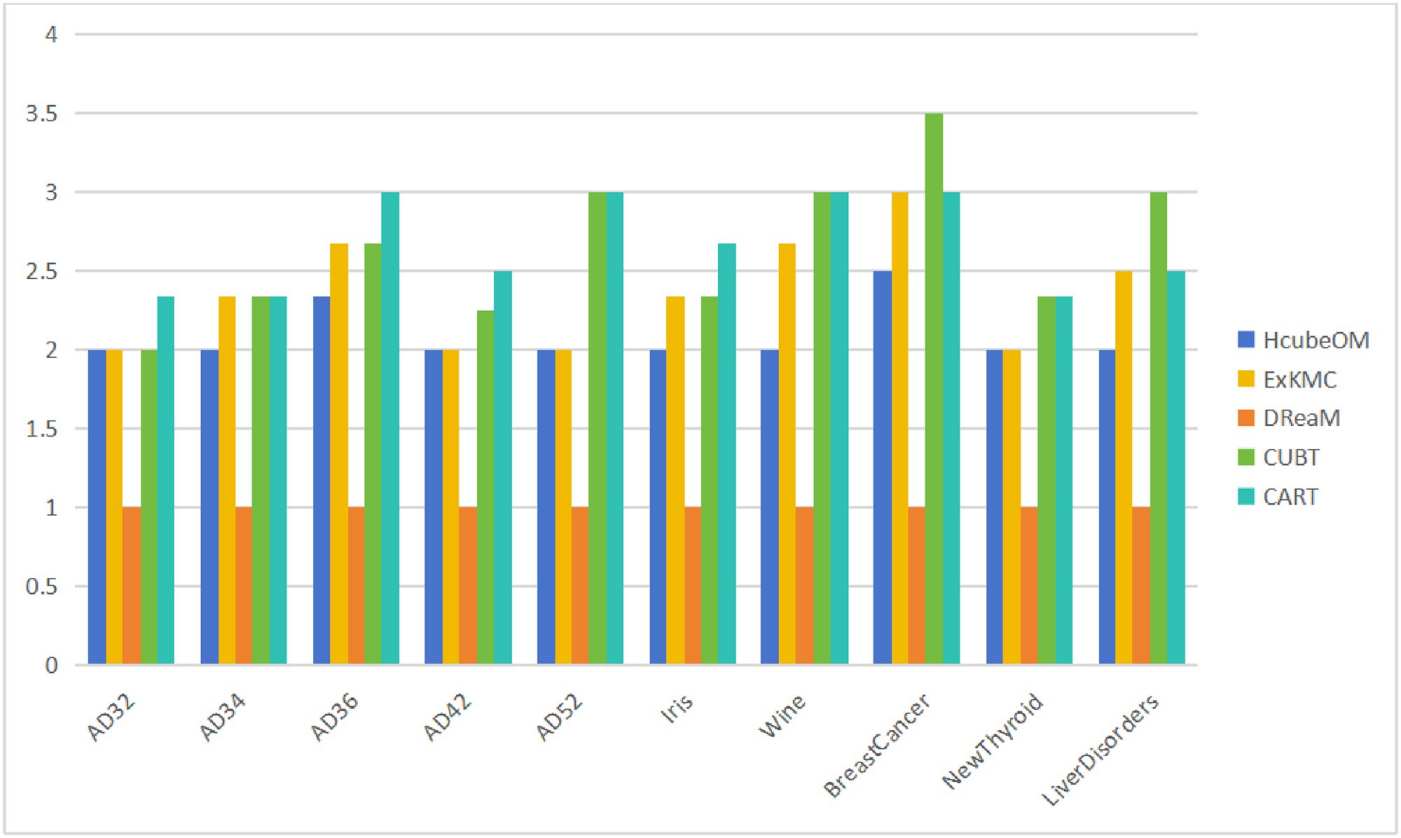
Average number of hypercubes. The average number of hypercubes obtained by each method in each dataset.

The average number of hypercubes obtained by each method in each dataset is calculated in the experiments and its used to illustrate the validity of the SR metrics. The specific values are shown in Fig 17. The most important contribution of DReaM is that the decision boundaries of each cluster can be adjusted based on priori knowledges, but the division between clusters is still based on a hypercube, so the average number of hypercubes is 1 on all datasets. The results of DReaM can be considered as a special case and are not analyzed in comparison with other methods in terms of the average number of hypercubes.

Observing the results of the other four methods in Fig 17, the average number of hypercubes of HcubeOM is less than that of ExKMC, CUBT, and CART, which suggests that HcubeOM is easier to generate concise explanations than ExKMC, CUBT, and CART, and also corresponds to the results in Table 5.

Combining the results of the analysis of the Table 5 and Fig 17 illustrates that HcubeOM can generate more concise explanations and have good robustness.

### The consistency of explanations

**Fig 18:**
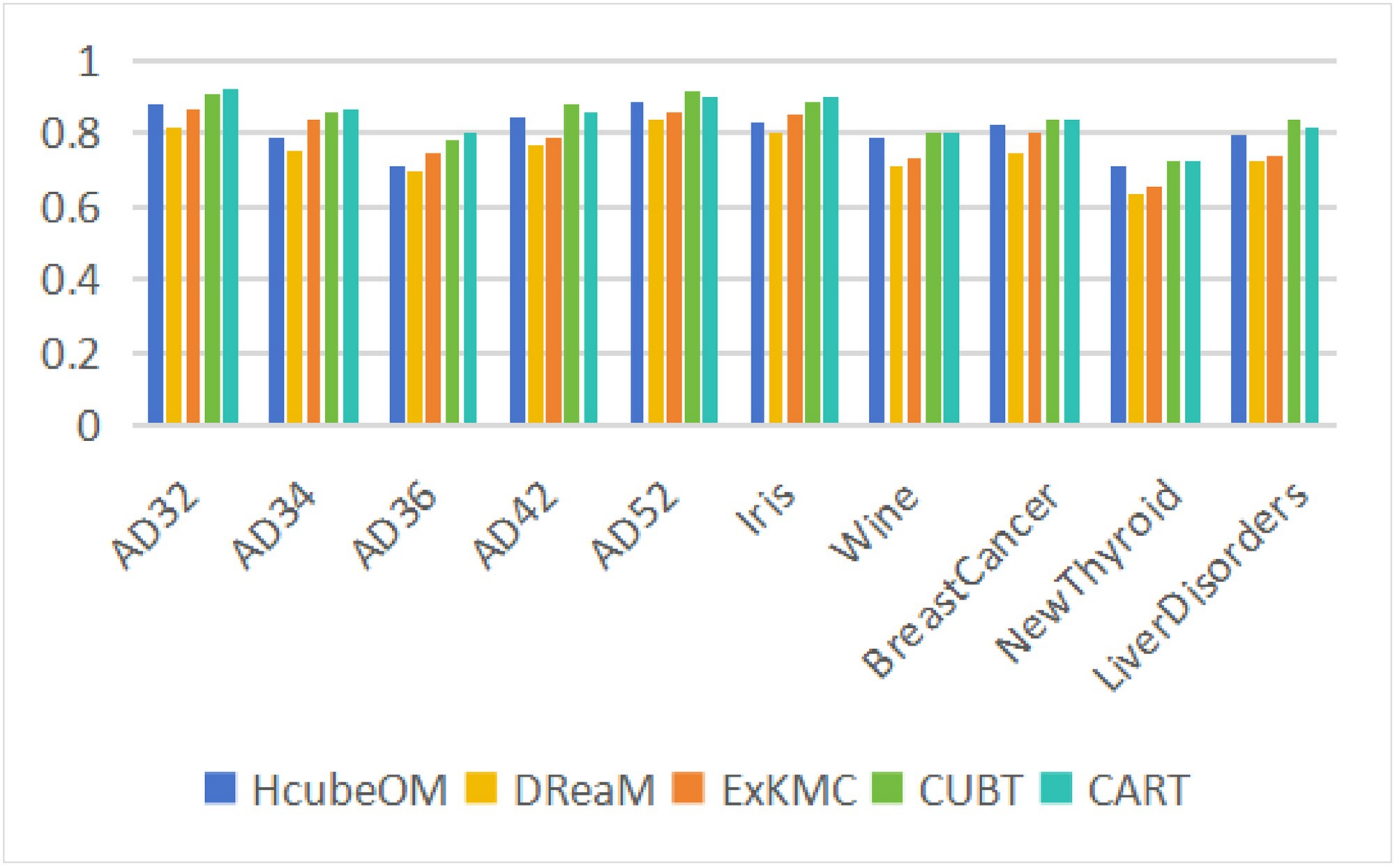
TPR values. The TPR values of different methods.

In this section, the performance of each method in terms of explanations consistency is verified. The values of ER and TPR are calculated by HcubeOM and other comparison methods. In order to reflect the robustness of the experimental results, each method is run 15 times on each dataset, and finally the mean and standard deviation of the 15 results are computed as the final comparison. Table 6 is the specific calculation results, each metric is marked with ↑ or ↓ to indicate whether it should be maximized or minimized. To facilitate the observation of changes in indicator values, we use bar charts to show the TPR values for different methods, see Fig 18.

**Table 6:** Average ER and TRP values on different methods are compared, in which those in brackets are the corresponding standard deviations.

Dataset	HcubeOM		ExKMC		CUBT		CART		DReaM	
	ER↓	TPR↑	ER↓	TPR↑	ER↓	TPR↑	ER↓	TPR↑	ER↓	TPR↑
AD32	0.05365 (0.04584)	0.88162 (0.04449)	0.05568 (0.04636)	0.86549 (0.04584)	0.04263 (0.07973)	0.90976 (0.08796)	**0.04056** (0.02412)	**0.91876** (0.02570)	0.05874 (0.06674)	0.81549 (0.05614)
AD34	0.06558 (0.04701)	0.79003 (0.04611)	0.06361 (0.04683)	0.83390 (0.04201)	0.05256 (0.09087)	0.85817 (0.08632)	**0.05049** (0.02293)	**0.86717** (0.02698)	0.06867 (0.06328)	0.75390 (0.05343)
AD36	0.07254 (0.04630)	0.71088 (0.04565)	0.06857 (0.04044)	0.74476 (0.04330)	0.05952 (0.09109)	0.77903 (0.08214)	**0.05745** (0.02176)	**0.79803** (0.02532)	0.07563 (0.06597)	0.69475 (0.05909)
AD42	0.05069 (0.04553)	0.84370 (0.04293)	0.05272 (0.04775)	0.78757 (0.04553)	**0.04067** (0.08977)	**0.88184** (0.08438)	0.04261 (0.02551)	0.86084 (0.02831)	0.05678 (0.06692)	0.76757 (0.05577)
AD52	0.05118 (0.04534)	0.88561 (0.04449)	0.05321 (0.04719)	0.85949 (0.04834)	**0.04016** (0.09280)	**0.91375** (0.08211)	0.04209 (0.02614)	0.90275 (0.02681)	0.05627 (0.06373)	0.83948 (0.05575)
Iris	0.17523 (0.04663)	0.82811 (0.04077)	0.15726 (0.04044)	0.85198 (0.04063)	0.10221 (0.08895)	0.88625 (0.08770)	**0.08414** (0.02195)	**0.89955** (0.02367)	0.19932 (0.06391)	0.80198 (0.05688)
Wine	0.32537 (0.04742)	0.78587 (0.04049)	0.33940 (0.04778)	0.72975 (0.04771)	**0.28550** (0.08894)	**0.80402** (0.08675)	0.31635 (0.02821)	0.80262 (0.02357)	0.37246 (0.06696)	0.70974 (0.05749)
BreastCancer	0.23386 (0.04534)	0.81902 (0.04371)	0.23789 (0.04630)	0.80290 (0.04734)	**0.19399** (0.08527)	**0.83717** (0.09169)	0.21484 (0.02526)	0.83577 (0.02622)	0.26095 (0.06768)	0.74289 (0.05415)
NewThyroid	0.21390 (0.04532)	0.70815 (0.04227)	0.22793 (0.04601)	0.65203 (0.04532)	**0.17403** (0.08613)	**0.72629** (0.08793)	0.20488 (0.02771)	0.72489 (0.02740)	0.24099 (0.06600)	0.63202 (0.05815)
LiverDisorders	0.23830 (0.04421)	0.79675 (0.04492)	0.24233 (0.04481)	0.74062 (0.04421)	**0.20843** (0.08904)	**0.83489** (0.08535)	0.22928 (0.02821)	0.81349 (0.02895)	0.26539 (0.06628)	0.72062 (0.05834)

As can be seen from the bar charts and tables, the results of CUBT and CART are better than the other three methods. CART itself is a decision tree. CUBT is an improved method based on a decision tree, and there has a series of recursive binary splits are performed on the dataset. So their metric values are better than HcubeOM. However, the value of SR of HcubeOM is much better than that of CUBT and CART. The most important goal of this work is to provide succinct and acceptable explanations to the user, so a poor consistency of explanations is acceptable in the case of good SR values.

In the three datasets AD34, AD36 and Iris, the explanations consistency of HcubeOM is slightly worse than that of ExKMC, which is mainly due to the fact that the distribution of data in these three datasets is relatively discrete, so the hypercubes eventually obtained by HcubeOM will contain a lot of redundant regions, which has a bad impact on the consistency of the metric explanations. In the rest of the datasets, the consistency of the explanations of HcubeOM is better than that of ExKMC and DReaM. But the difference in the two metric values between ExKMC and HcubeOM is very little, this is mainly because HcubeOM is highly similar to ExKMC, and the expansion of *k* nodes to *k*′ nodes of the threshold tree in ExKMC is similar to the increase of the number of hypercubes in HcubeOM. In DReaM, the generation of explanations ultimately relies on a rectangular box. Therefore, there will be a large error in division of the raw dataset based on the obtained explanations. Overall, HcubeOM outperforms ExKMC and DReaM on most datasets, suggesting that our approach makes sense.

## Conclusion

In this paper, a method for explaining clustering results based on the hypercube overlay model is proposed. Two objective functions are used to find the optimal hypercube overlay scheme for each cluster. Then, by combining the hypercubes in each cluster, the explanations of each cluster can be obtained. Furthermore, a series of experiments and comparisons among HcubeOM and the known explanations methods show that HcubeOM provide a more concise and understandable explanations for users.

There are some shortcomings in our approach. First, the method treats each cluster as an optimization object and does not take into account the cluster-to-cluster effects. Second, the explanations obtained by the method is still only for the dataset itself and cannot be applied to specific practice areas. Third, we have only illustrated the validity of the simplicity metric SR in this work, and have not illustrated the generalizability of SR with extensive experiments. In the future we will consider inter-cluster effects to improve the proposed model, and explore methods to provide explanations in the clustering process. We will also verify the validity of the SR metrics in more clustering explanatory methods.
